# Functional characterization of miR-708 microRNA in telomerase positive and negative human cancer cells

**DOI:** 10.1038/s41598-021-96096-y

**Published:** 2021-08-23

**Authors:** Zeenia Kaul, Caroline T. Y. Cheung, Priyanshu Bhargava, Anissa Notifa Sari, Yue Yu, He Huifu, Hemant Bid, Jeremy D. Henson, Joanna Groden, Roger R. Reddel, Sunil C. Kaul, Renu Wadhwa

**Affiliations:** 1 Children’s Medical Research Institute, Faculty of Medicine and Health, The University of Sydney, Westmead, NSW 2145 Australia; 2 AIST-INDIA DAILAB, DBT-AIST International Center for Translational & Environmental Research (DAICENTER), National Institute of Advanced Industrial Science & Technology (AIST), Tsukuba, 305-8565 Japan; 3 The Ohio State Medical Comprehensive Cancer Center, 460 W 12th Avenue, Columbus, OH 43210 USA; 4 Biomedical Research Institute, National Institute of Advanced Industrial Science and Technology (AIST), 1-8-31, Ikeda, Osaka, 563-8577 Japan; 5 University of Illinois, 310 AOB MC 672, Chicago, IL 60607 USA; 6 Life Sciences Institute, University of Michigan, Ann Arbor, MI 48109 USA; 7 Cancer Cell Immortality Group, Prince of Wales Clinical School, University of New South Wales, Sydney, NSW 2052 Australia

**Keywords:** Cancer, Cancer prevention

## Abstract

Activation of a telomere length maintenance mechanism (TMM), including telomerase and alternative lengthening of telomeres (ALT), is essential for replicative immortality of tumor cells, although its regulatory mechanisms are incompletely understood. We conducted a microRNA (miRNA) microarray analysis on isogenic telomerase positive (TEP) and ALT cancer cell lines. Amongst nine miRNAs that showed difference in their expression in TEP and ALT cancer cells in array analysis, miR-708 was selected for further analysis since it was consistently highly expressed in a large panel of ALT cells. miR-708 in TEP and ALT cancer cells was not correlated with C-circle levels, an established feature of ALT cells. Its overexpression induced suppression of cell migration, invasion, and angiogenesis in both TEP and ALT cells, although cell proliferation was inhibited only in TEP cells suggesting that ALT cells may have acquired the ability to escape inhibition of cell proliferation by sustained miR-708 overexpression. Further, cell proliferation regulation in TEP cells by miR708 appears to be through the CARF-p53 pathway. We demonstrate here that miR-708 (i) is the first miRNA shown to be differentially regulated in TEP and ALT cancer cells, (ii) possesses tumor suppressor function, and (iii) deregulates CARF and p21^WAF1^-mediated signaling to limit proliferation in TEP cells.

## Introduction

Telomeres are specialized structures located at the ends of linear chromosomes of eukaryotic cells, and they provide several very important biological functions. They prevent the natural chromosome ends from being recognized as DNA breaks and are safeguarded by a capped conformation of specific telomere-associated proteins and DNA tandem repeats, thus preserving genetic material and stability as well as inhibition of aberrant DNA damage response activation^1,2^. Telomeres play a major role in structural and functional organization of chromosomes^3^, regulation of gene expression^4^ and the replicative senescence (Hayflick limit)^5^ of normal cells. In normal cells, telomeres gradually shorten with each cell division which eventually results in replicative senescence^6^. In contrast, cancer cells activate a telomere length maintenance mechanism (TMM), resulting in cellular immortalization, which is a critical step towards malignancy. The most common TMM which is activated in approximately 85–90% of tumors is the telomerase enzyme, the core components of which are an RNA component (hTR/hTERC) and a catalytic protein component with reverse transcriptase activity, hTERT^5,7^. The RNA component binds to the 3′ telomeric sequences and acts as a template for synthesis of new telomeric repeats by reverse transcription^8^. On the other hand, ALT (Alternative mechanism of Lengthening of Telomeres) is observed in 10–15% of cancers, but with a very high occurrence in certain types of tumors, such as sarcomas and astrocytomas^9^. ALT consists of a telomerase-independent and homologous recombination (HR)-mediated mechanism to expand telomeres, in which existing telomeric DNA is used as a template for synthesis of new telomeric repeats^10,11^. ALT is further characterized by the presence of unique ALT-associated promyelocytic leukemia protein nuclear bodies (APBs) that encompass abundant amounts of extrachromosomal telomeric DNA, such as partly single-stranded circles of telomeric DNA in which the cytidine-rich strand is intact (C-circles), as well as telomere-associated and HR proteins^12–14^. However, the underlying mechanisms for activation of telomerase and ALT, especially their regulators and the determinants for selection of TMM method in different tumor types, and the consequences for tumor cell characteristics, are still only partly understood.

Recent studies have indicated that microRNAs (miRNAs), 20–24 base pair non-coding RNAs that silence target genes at the post-transcriptional level by binding to complementary sequences in the 3′UTR to promote transcript degradation or translation inhibition, play major roles in the regulation of cancer^15,16^. They have been reported to be involved in regulation of cell cycle progression, DNA damage response, apoptosis, epithelial-to-mesenchymal cell transition, cell motility/invasion, stemness, and also in telomere maintenance^17–19^. It was recently shown that miR-380-5p impaired telomerase activity in peritoneal mesotheliomas, but unexpectedly, ALT was activated in spite of inhibition of cancer growth^20^. Further, miR-23a was demonstrated to induce telomere shortening by downregulating TRF2 (telomeric repeat binding factor 2), an essential telomeric protein that not only keeps the telomere intact, but can also associate with various proteins involved in telomere assembly, length regulation, DNA replication, repair, recombination, and cell cycle control^21^. However, a majority of these studies have focused on the regulation of telomerase-dependent TMM by miRNAs, and none has investigated the differences in miRNA regulation between TEP and ALT cells. Since telomere maintenance is an essential aspect of attaining cellular immortality, it is important to gain further understanding of the mechanisms governing the two TMMs, which could potentially facilitate devising of future cancer therapeutic strategies. In this study, we aimed to examine the differences in miRNA species between TEP and ALT tumor cells in order to shed light on their role in these two TMMs. In order to narrow-down the differences in miR-expression patterns that may emerge from the heterogenic nature of cancer cells, we used isogenic cells (expressing either telomerase or ALT related mechanisms of telomere lengthening^22^) for microarray. The selected miRNA species were investigated in a large panel of TEP and ALT cells following which the biological significance of the selected miRNA (miR-708) in pathways related to the regulation of telomere length, DNA damage, cell proliferation and migration was determined in representative TEP (MG63) and ALT (U2OS) by cellular, biochemical and expression analyses.

## Materials and methods

### Cell lines and culture

Human normal (Fre102s-3 and WI38) and cancer cells possessing either TEP (MG63, 293T, JFCF-6/T.1J/6B, JFCF-6/T.1C, JFCF-6/T.1J/9E, JFCF-6/T.1F, GM639, MRC-5V1, F80-T2b, SJSA-1, TE85, A549), or ALT (U2OS, SaOS2, VA13, JFCF-6/T.1J/1-3C, JFCF-6/T.1J/5H, MRC-5V2, GM847, G292, IIICF/c) mechanisms of telomere lengthening were cultured in Dulbecco’s modified Eagle’s medium (DMEM; Gibco BRL, Grand Island, NY, USA) supplemented with 10% fetal bovine serum (Gibco BRL), penicillin (100 IU/mL), and streptomycin (50 µg/mL) in the presence of 5% CO_2_ at 37 °C. JFCF-6 strains were derived from SV40-transformation of mortal jejunal parental fibroblasts (JFCF-6) from a male cystic fibrosis patient as described earlier^22^. The MRC-5 cell strains were obtained from SV-40 transformed human diploid fibroblasts described earlier^23^. Other immortalized cell lines were purchased from the American Type Culture Collection (Manassas, VA, USA) and confirmed to be mycoplasma free. miR-708 overexpressing and compromised cells were generated using vectors (pEP-miR; from Cell Bio labs, Inc) containing EF1-α promoter. miR708 stem loop precursor (114 bp) (5′-CUGUGUGUGAAGUGGUAACUGCCCUC***AAGGAGCUUACAAUCUAGCUGGG***GGUAAACGACUUGCACAUGAA***CGCAUCUAGACUGUGAGCUUCUAGA***GGGCAGGGACCUUACCCUA-3′) flanked by a human intron sequence was used to preserve its hairpin structure and endogenous processing. miR-708 sequence was replaced by ***UUCUCCGAACGUGUCACGUTT*** and ***ACGUGACACGUUCGGAGAATT*** in control vector. Cells were transfected using Lipofectamine 2000 (Invitrogen) and selected in puromycin (10 μg/mL) supplemented medium.

### microRNA microarray

Small RNAs that were < 200 nucleotides in length were extracted from normal (Fre102s-3), TEP (JFCF-6/T.1J/6B), and ALT (JFCF-6/T.1J/1-3C) cells using the mirVana miRNA isolation kit (Ambion, Austin, TX, USA) as per the manufacturer’s protocol. The purified RNA was labeled with Cy dyes, hybridized to array slides containing 817 human miRNAs (Hokkaido System Science, Japan), and detected by a scanner (Agilent Technologies, Santa Clara, CA, USA) as described earlier^24^. The miRNA species that were differentially expressed by at least fivefold between TEP and ALT cells were selected for further analysis.

### Reverse transcription quantitative PCR (RT-qPCR)

Small RNAs were extracted using the mirVana miRNA isolation kit (Ambion), then reverse-transcribed, and the qPCR reactions were performed using a Taqman Small RNA Assay kit with gene-specific primers. The results were normalized to the small nucleolar RNA, RNU6B. For detection of mRNA transcripts, total RNA was purified using the RNeasy plus mini kit and 500 ng of RNA was reverse transcribed with the QuantiTect Reverse Transcription kit (both Qiagen, Hilden, Germany) as per the manufacturers’ protocol. qPCR was performed with 1/25 diluted cDNA using the SYBR Select Master Mix kit (Applied Biosystems, Foster City, CA, USA) on the EcoTM Real-time System (Illumina, San Diego, CA, USA) and normalized to GAPDH as previously described^25^.

### C-circle assay and telomere length assay

C-circles were detected by rolling circle amplification^11^ and then visualized by either telomere qPCR or slot–blot analysis as previously described^26^. To determine telomere length, telomere qPCR was performed for each sample, and the difference in the cycle threshold (Ct) between a telomere-specific PCR reaction and a single copy gene (SCG) PCR reaction was calculated for each sample as the ΔCt^26^, which represented the average relative telomere repeat length.

### Telomerase activity detection (TRAP assay)

A telomeric repeat amplification protocol (TRAP) enzyme-linked immunosorbent assay (ELISA) kit (Roche, Mannheim, Germany) was used as per the manufacturer’s protocol to determine telomerase activity as previously described^27^.

### miR target prediction

Four different online programs, miRDB, TargetScan, mirtarbase, and miRmap, were used to search for potential targets of miR-708 as per the programs’ protocols.

### Luciferase reporter assay

The pMIR-CARF-3′UTR plasmid was cloned and used as previously described^25^. The stably transfected miR-335-overexpressing cells were used as positive controls as described in previous studies^25,28^. An equal number of stably transfected miR-708-overexpressing, miR-708-deficient, and control cells were plated in 24-well plates. Cells were transfected with 1 μg of the luciferase constructs (BRCA1-3′UTR, MRE11-3′UTR) (Life Technologies, Invitrogen) or pMIR-CARF-3′UTR) and 100 ng of control vector oligonucleotide (pRL-TK or pMIR-REPORTTM β-gal control plasmid) using X-remeGENE HPDNA transfection reagent (Roche Applied Science, Penzberg, Germany). Cells were harvested at 70% confluency followed by quantitation of luciferase activity using the Dual Luciferase Reporter Assay System and the Infinite M200 Pro Microplate Reader (Tecan, Mannedorf, Switzerland) as per the manufacturer’s instructions.

### Tube formation assay

The formation of capillary-like structures on a basement membrane matrix of human umbilical vessel endothelial cells (HUVECs) was used to assess the angiogenesis promoting activity of miR-708. Culture wells (16-mm diameter) were coated with 250 μL of Matrigel (BD Biosciences, Franklin Lakes, NJ, USA) for 30 min at 37 °C. Then, HUVECs were serum-starved in serum-free EGM-2 (endothelial cell growth medium-2) for 6 h, harvested, and suspended in conditioned media that contained 50 ng/mL VEGF165. Subsequently, the HUVECs were treated with the conditioned media from miR-708-overexpressing, miR-708-deficient, or control cells at a density of 1 × 10^5^ cells/well. The treated cells were then seeded into the Matrigel-coated wells and allowed to form vesicular tubes for 24–72 h at 37 °C. Tube formation was photographed at a magnification of 50X. The area covered by the tube network was quantified by Image-Pro Plus software (Media Cybernetics, Silver Spring, MD, USA).

### Western blotting

Cells were lysed in RIPA lysis buffer in the presence of protease inhibitors (Roche), and protein concentration was determined using the BCA Protein Assay kit (Bio-Rad, Hercules, CA, USA). Equal protein amount from each sample (20–50 μg) was separated by SDS–polyacrylamide gel electrophoresis, and then electroblotted onto polyvinylidene fluoride (PVDF) membranes using a semi-dry transfer system (ATTO, Amherst, NY, USA). The protein membranes were blocked in TBS-T containing 5% skim milk, incubated with anti-MRE11 (NB-100-142) (Novus Biological), anti-BRCA1 (CST 9010) (Cell Signaling Technology), anti-CARF^28^, anti-ATR (ab4471) (Abcam), anti-ATM (2C1) (SC23921, Santa Cruz Biotechnology), anti-ERK2 (C-14) (SC154,Santa Cruz Biotechnology), anti-p53 (DO-1) (sc-126; Santa Cruz Biotech, Santa Cruz, CA, USA), and anti-p21 (C-19) (sc-397; Santa Cruz Biotech) antibodies. Actin was used as an internal loading control (anti-actin antibody, Chemicon, Burlington, MA, USA). The blots were subsequently incubated with HRP (horse radish peroxidase)-conjugated anti-mouse or anti-rabbit secondary antibody (sc-2004 or sc-2005; Santa Cruz, Santa Cruz, CA, USA). Finally, the blots were visualized using enhanced chemiluminescence (ECL) (GE Healthcare, Chicago, IL, USA).

### Wound healing migration and in vitro cell invasion assays

For the wound healing assay, a pipette tip was used to scratch straight through cells grown in monolayer to create a "wound", followed by PBS washes to remove cell debris and addition of fresh medium. The time of wound creation was designated as 0 h. Subsequently, the cells were allowed to grow and migrate into the wound for a minimum of 36 h, which was recorded using a phase contrast microscope with a 10× phase objective lens.

An in vitro cell invasion assay was performed by seeding 5 × 10^4^ cells into the upper chamber of 16‐well CIM plates with 8 μm pores (xCELLigence System, ACEA Biosciences, San Diego, CA, USA), which was coated on the surface with a 1/10 dilution of Matrigel (BD BioSciences). The cells were allowed to migrate and invade into the lower chamber for up to 24 h, and then the invading cells were fixed, stained with Crystal violet, and counted under phase contrast microscopy. The graphs are shown as the % of cells that had invaded into the Matrigel as compared to the number of plated cells.

### Cell proliferation and colony-forming assays

The effect of miR-708 on cell proliferation was determined by direct cell counting and by measuring the conversion of the tetrazolium salt, 3-(4,5-dimethyl-thiazol-2-yl)-2,5-diphenyltetrazo-lium bromide (MTT), to formazan. Briefly, 5000 stably transfected miR-708-overexpressing, miR-708-deficient, and control cells were plated in 96-well plates, and after 2 days of incubation at 37 °C, 200 µL of MTT (Sigma Chemical Corp., St. Louis, MO, USA) in PBS (2 mg/mL) was added to each well. After 4 h of incubation at 37 °C, the medium was removed and 100 µL of dimethyl sulfoxide (DMSO) was added. Plates were then read on a microplate reader at 540 nm.

Colony-forming assays were performed to determine long-term tumorigenic effects. Five hundred stably transfected miR-708-overexpressing, miR-708-deficient, or control cells were plated into the wells of a 6-well plate and allowed to proliferate at 37 °C for 10–15 days with regular changes of medium every third day. The plates were then fixed with pre-chilled methanol/acetone (1:1, v:v) for 10 min on ice, stained with 0.1% crystal violet solution, scanned, and counted.

### Cell-cycle analysis

TEP and ALT cells were seeded and harvested, then centrifuged at 2000 rpm, 4 °C, for 5 min. Cells pellets were washed with cold PBS and fixed with 70% ethanol. The fixed cells were stored at − 20 °C for 24 h or until further use. The fixed cells were centrifuged at 3000 rpm, 4 °C, for 10 min, washed with cold PBS twice, then re-suspended in 1 mL cold PBS and were added with RNase A (1 mg/mL at 37 °C for 30 min). Guava Cell Cycle Reagent (4500-0220) (Luminex Corporation, Austin, TX, USA) was used to stained cells in the dark for 30 min. Cell-cycle results were analyzed using Guava® PCA-95 System (Luminex Corporation, Austin, TX USA) and flow cytometry data was analyzed using FlowJo software (Version 7.6, Flow Jo, LLC, Ashland, OR, USA).

### Statistical analyses

GraphPad Prism 8 was used to create graphs and perform statistical analyses using unpaired Student’s two‐tailed *t*‐tests. Significance was determined when the *p* value < 0.05.

## Results

### Identification of differentially regulated miRNAs in TEP and ALT cells by microarray

**Figure 1:**
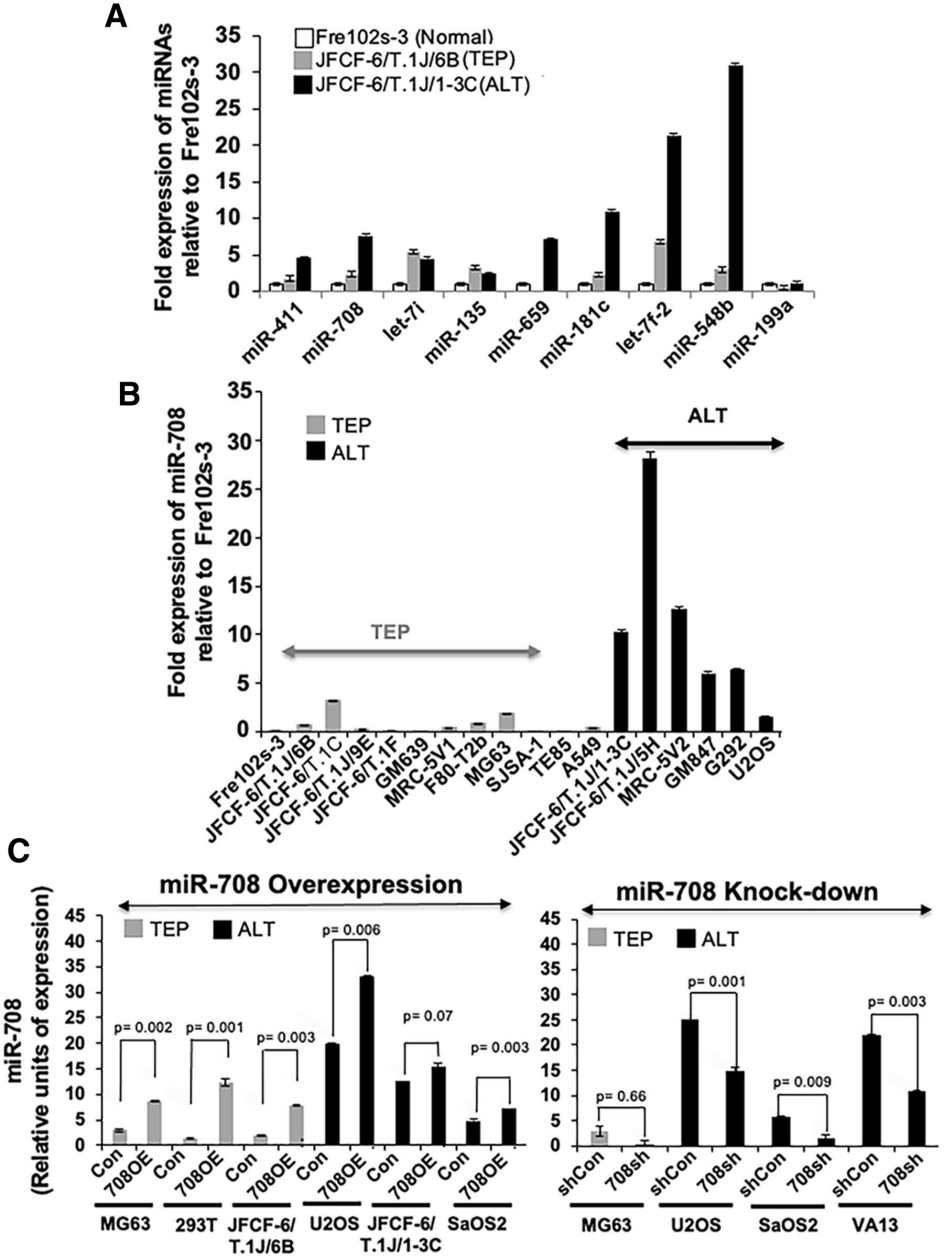
Identification of miRNAs differentially expressed in TEP and ALT cells. (**A**) Quantitative real-time PCR (qPCR) validation of the differentially expressed nine miRNAs that showed at least fivefold difference in expression in the miRNA array between isogenic TEP (JFCF-6/T.1J/6B) and ALT (JFCF-6/T.1J/1-3C) cells. (**B**) Comparison of miR-708 levels between various TEP and ALT cells by qPCR. miRNA expression is shown as fold expression as compared to control, normal (Fre102s-3) cells. The bars depicting normal cells are shown in white, TEP cells are shown in grey, and ALT cells are shown in black. Data represent mean ± SD (n = 3). A variety of TEP and ALT cells were used for miR-708 overexpression and knockdown of miR-708. (**C**) qPCR for miR-708 was performed in control, miR-708-overexpressing, and miR-708-deficient TEP and ALT cells. Data represent mean ± SD (n = 3). Con: control for miR-708 expression; 708OE: miR-708 expression; shCon: control for miR-708 knock-down; 708sh: miR-708 knock-down using short-hairpin interfering RNA. *p* < 0.05 was considered as statistically significant.

Isogenic TEP (JFCF-6/T.1J/6B and JFCF-6/T.1J/6G) and ALT (JFCF-6/T.1J/1-3C and JFCF-6/T.1J/1-4D) cells were subjected to microarray analysis to determine differences in expression of miRNAs. Microarray data showed 515 genes differentially expressed in at least one of the cell lines (Supplementary Fig. 1A). Of note, there were not that many miRNA genes that were consistently expressed in a similar manner in ALT versus TEP cells. Around 300 miRNAs were either not expressed, or not differentially expressed amongst all TEP and ALT cells. Amongst these, we selected nine candidate miRNAs (miR-135, miR-181c, miR-199a, miR-411, miR-548b, miR-659, miR-708, let-7f.-2, and let-7i) that had at least fivefold difference in their expression between the TEP and ALT cells. For further validation, expression of the selected miRs was examined by RT-qPCR in isogenic normal fibroblasts (Fre102s-3), and their TEP (JFCF-6/T.1J/6B) or ALT (JFCF-6/T.1J/1-3C) immortal derivatives. As shown in Fig. 1A, we found that the expression level of miR-411, miR-708, miR-659, miR-181c, let-7f.-2, and miR-548b was higher in ALT cells as compared to TEP and normal fibroblasts (Fig. 1A). Among these six RT-qPCR-validated miRNAs, miR-708 was further confirmed to be predominantly overexpressed in ALT cells in a large panel of isogenic and non-isogenic TEP and ALT cell lines. Similar to Fre102s-3, WI38 normal fibroblasts showed a low level of expression (Fig. 1B and Supplementary Fig. 1B). Based on these data, we selected miR-708, as one of the miRs with higher level of expression in ALT cells, for further analyses in this study. In order to determine the functional role of miR-708 in ALT and TEP tumor cells, we performed genetic manipulation via stable overexpression and knock-down of miR-708 using specific vectors in various TEP and ALT cell lines. Figure 1C shows the stable cell lines in which miR-708 overexpression (OE) or knock-down of miR-708 were successfully obtained by respective vectors. These cells (at PD21-25) were used for further analysis.

### Functional characterization of miR-708 for TEP and ALT cell characteristics

**Figure 2:**
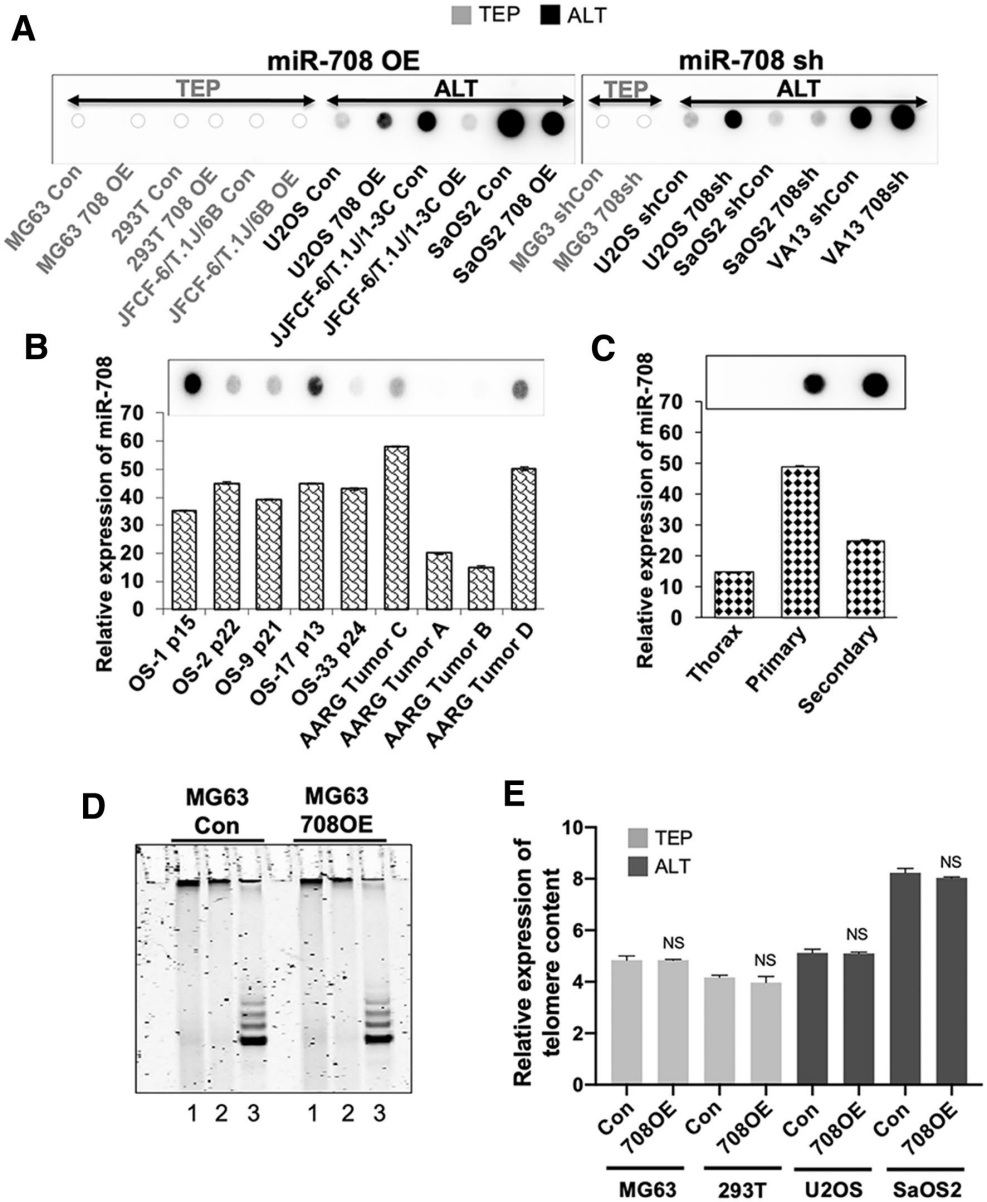
Effects of miR-708 on C-circles, telomerase activity and telomere length. (**A**) Slot blot analysis of C-circle content of miR-708-overexpressed (OE) and -deficient (sh) TEP and ALT cells, showing that miR-708 depletion increases C-circle content in 3/3 ALT cell lines and overexpression decreases C-circle content in 2/3 ALT cell lines. As expected, C-circles were not detected in TEP cell lines. (**B**) miR-708 expression in TEP and ALT osteosarcoma tumor samples. Top panel—Slot blot C-circle assay in pediatric patient derived xenografts (OS-1, OS-2, OS-9, OS-17 and OS-33) and adult osteosarcoma tumors (AARG). Lower panel—quantitation of miR-708 expression; data represent mean ± SD values compared within the group. (**C**) Top panel—Slot blot C-circle assay in normal thoracic tissue, and in primary and secondary tumors from a pediatric patient with osteosarcoma; lower panel—quantitation of miR-708 expression. (**D**) Telomerase activity as determined by the TRAP assay in control and miR708-overexpressed MG63 cells. Lane 1 shows water control, lanes 2 and 3 show TRAP assay with and without heat inactivation of telomerase, respectively. (**E**) Telomere qPCR was performed to determine content of telomeric DNA, expressed in arbitrary units (a. u.) in miR-708-overexpressed TEP and ALT cells. NS: not significant when comparing control and miR-708-overexpressed cells. Con: control for miR-708 expression; 708OE: miR-708 expression; shCon: control for miR-708 knock-down; 708sh: miR-708 knock-down using short-hairpin interfering RNA. NS => 0.05

In order to investigate whether the overexpression of miR-708 was associated with TMM, we first examined the association of miR-708 with C-circles, an established assay for ALT cells, in miR-708-overexpressing and -deficient cell lines. As shown in Fig. 2A, overexpression of miR-708 caused a decrease in C-circles in two (JFCF6/T.1J/1-3C and Saos2/708), and an increase in one (U2OS) ALT cell line. On the other hand, miR-708 knockdown with siRNA caused an increase in C-circles in three of three ALT cell lines (U2OS, Saos2 and VA13). Taken together, the data suggested that, although miR-708 is highly expressed in most ALT cells (Fig. 1B), it did not show any correlation with C-circle levels that are considered as consistent markers for ALT cells.

We next explored the association of miR-708 with C-circle levels in clinical samples of osteosarcomas, a tumor type in which ALT is common^14^. As shown in Fig. 2B, seven of the nine tumors were ALT-positive as indicated by the presence of C-circles. Of note, in these seven (five pediatric patient derived xenografts (OS-1, OS-2, OS-9, OS-17 and OS-33) and two adult osteosarcoma tumor (AARG) samples, the miR-708 levels were clearly higher as compared to the two C-circle-negative tumors. However, amongst the ALT tumors, there was no obvious correlation between the level of miR-708 expression and C-circle abundance. Similarly, an examination of normal, the primary osteosarcoma, and a metastasis tissues from the same individual showed that the normal tissue was C-circle-negative, as expected, whereas the primary and secondary tumor both were C-circle positive; the normal tissue had an miR-708 expression level which was clearly lower than the tumors (Fig. 2C). We also determined if miR-708 plays a role in telomerase activity in TEP cells. The TRAP assay detected no difference in telomerase activity between control and miR-708-overexpressing MG63 cells (Fig. 2D). Furthermore, assessment of telomere content in both TEP and ALT cells with miR-708 overexpression showed that there was no difference despite the altered miR-708 status. (Fig. 2E) These findings collectively demonstrate that miR-708 is consistently upregulated in ALT-positive cell lines and cancers compared to TEP/C-circle-negative cell lines and tissues, but its level does not influence telomerase activity, and it does not have correlation to C-circle levels in ALT cells.

### miR-708 targets DNA damage response genes and regulated CARF signaling differentially in TEP and ALT cells

**Figure 3:**
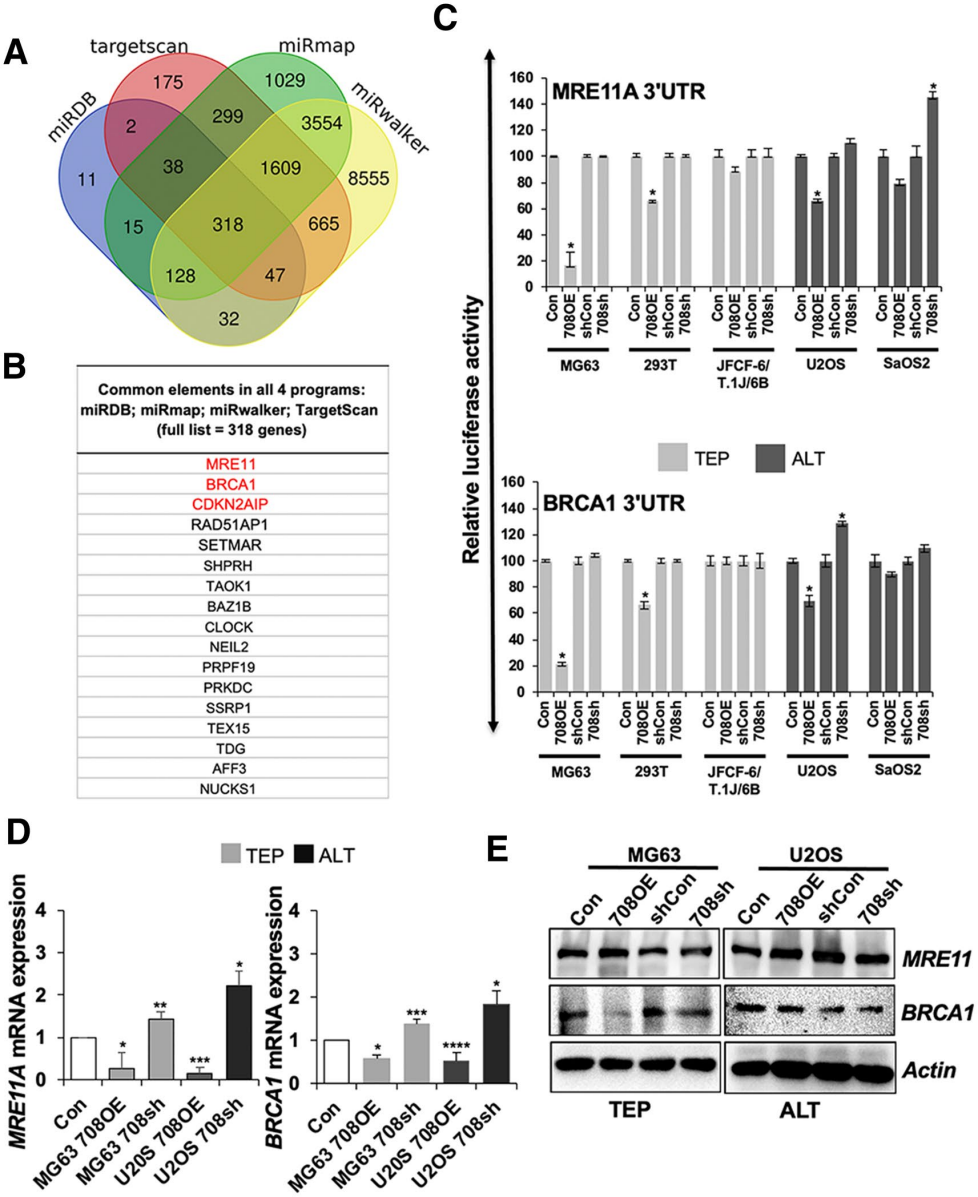
Prediction and validation of miR-708 targets. (**A**) Venn diagram showing the number of genes identified as potential targets of miR-708 as predicted by four algorithms: miRDB, Targetscan, miRmap, miRwalker. 318 genes were found to be common to all four programs. (**B**) MRE11A, BRCA1, and CDKN2AIP (CARF) were found by all four programs to be potential targets of miR-708. (**C**) Luciferase assays showing the repression of the 3′UTR of the candidate genes, MRE11A and BRCA1, by miR-708 in miR-708-overexpressing and -deficient TEP and ALT cell lines, compared to the relevant controls which were assigned a value of 100. Data are shown as mean ± SD, n = 4. (**D**) RT-qPCR validation of *MRE11A* and *BRCA1* transcript levels in miR-708-overexpressing and -deficient TEP and ALT cell lines, compared to the relevant controls (Con), which were assigned a value of 1. (**E**) Western blotting showed that miR-708 overexpression did not affect MRE11A protein levels, but decreased BRCA1 protein levels. Con: control for miR-708 expression; 708OE: miR-708 expression; shCon: control for miR-708 knock-down; 708sh: miR-708 knock-down using short-hairpin interfering RNA. *p < 0.05; **p < 0.01; ***p < 0.001; ****p < 0.0001.

In order to determine potential targets of miR-708, we used four different online programs, miRDB, TargetScan, mirtarbase, and miRmap, as shown in Fig. 3A,B. Amongst the list of 318 common candidate targets, MRE11A, BRCA1, and CDKN2AIP (commonly known as CARF) were especially interesting because they belong to the DNA damage response machinery that in turn regulates telomere function/dysfunction^29^. Of note, no other telomere-related genes were predicted as targets in this analysis. The MRN complex (MRE11A, RAD50, and NBS1) and BRCA1 are well-known as major regulators of the DNA damage response and repair pathways^30^. Based on these data, and instead of the further predictive analysis on the binding strength of miR-708 to these targets, we undertook the experimental analyses and next investigated the effect of miR-708 on these DNA damage response proteins using 3′UTR-luciferase reporter constructs. As shown in Fig. 3C, we found that miR-708 overexpression caused suppression of MRE11A- and BRCA1-3′UTR-driven luciferase expression in most TEP and ALT cells. These results were confirmed at the transcript level. Consistent to the reporter assay, MRE11A and BRCA1 mRNA expression was decreased in the miR-708 overexpressing cells (Fig. 3D). On the other hand, miR-708 knock-down did not show a significant effect in MRE11A- and BRCA1-UTR driven luciferase reporter assays in either TEP or ALT cell lines (Fig. 3C). mRNA expression analysis showed increase in MRE11A and BRCA1 in miR-708 depleted cells (Fig. 3D). At the protein level, in both TEP and ALT cells, miR-708 overexpression caused a decrease in BRCA1, but not MRE11A. However, neither MRE11A nor BRCA1 protein was differentially expressed between the TEP and ALT cells (Fig. 3E). Furthermore, miR-708 knock-down did not cause an increase in either of these two proteins in MG63 (TEP) or U2OS (ALT) cells (Fig. 3E and Supplementary Fig. 2A). Together, these data suggested that miR-708 targets MRE11A and BRCA1 in both TEP and ALT at the transcriptional level, however their expression at the protein level is most likely regulated by additional factors.

**Figure 4:**
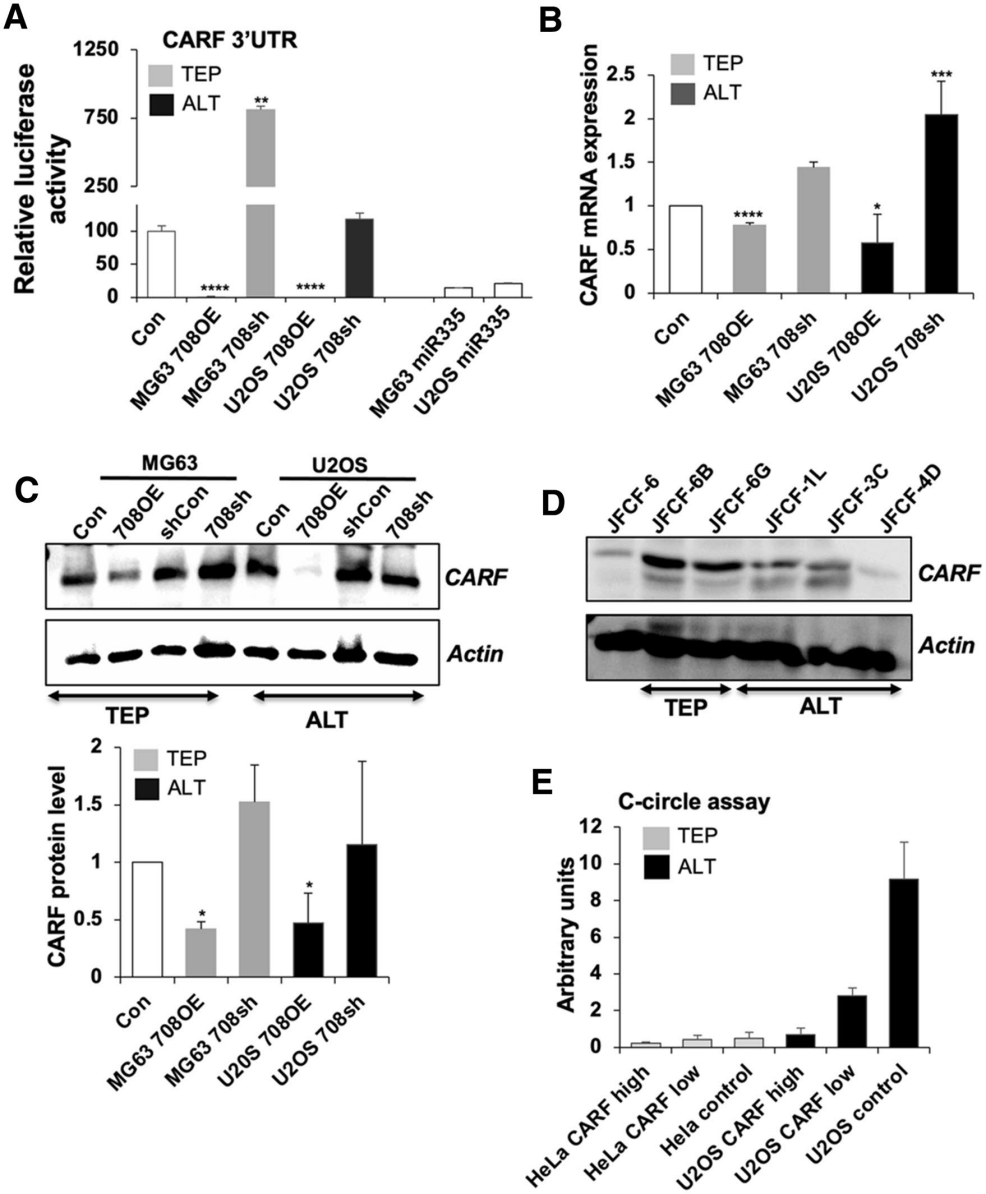
CARF is a target of miR-708 and is associated with C-circles. (**A**) Luciferase assay showing repression of CARF 3′UTR enhancer activity by miR-708 in miR-708-overexpressing and -deficient TEP and ALT cell lines. The relevant controls (Con) were assigned a value of 100. miR-355 overexpressing TEP and ALT cells were used as positive controls. (**B**) RT-qPCR for CARF shows that its transcript level was decreased in both miR-708-overexpressing MG63 and U2OS cells whereas it is increased in the miR-708-depleted cells. The relevant controls (Con) were assigned a value of 1. (**C**) Overexpression of miR-708 reduced CARF protein levels. Quantitation is shown in the lower panel as mean ± SD, n = 4. (**D**) In a panel of TEP (JFCF-6/T.1J/6B, JFCF-6/T.1J/6G) and ALT (JFCF-6/T.1J/1L, JFCF-6/T.1J/3C and JFCF-6/T.1J/4D) cell lines derived from the same parental cell line JFCF-6), the CARF protein level was lower in ALT cells than in TEP cells. (**E**) The amounts of C-circles in ALT cells-stably expressing extremely high (high) and moderately high (low) level of CARF as compared to the control (vector-infected cells). *p < 0.05; **p < 0.01; ***p < 0.001; ****p < 0.0001.

We next examined CDKN2AIP/CARF, which was another candidate target protein predicted by the four online programs. CARF is not only a regulator of the DNA damage response pathway, it is also a major modulator of the p53/p21^WAF1^ pathway that is responsible for cell fate determination, such as growth arrest, cell proliferation, malignant transformation and epithelial–mesenchymal transition (EMT)^29–32^. In order to determine if CARF is a direct target of miR-708, we performed luciferase reporter assays using 3′ UTR of CARF and found that overexpression of miR-708 caused strong inhibition of CARF-3′ UTR driven luciferase (*p* < 0.0001), while suppression of miR-708 induced a remarkable increase in luciferase activity (*p* = 0.0029) (Fig. 4A). Furthermore, analysis of CARF mRNA in miR-708-overexpressing and -compromised cells supported that miR-708 targets CARF in both TEP and ALT cells. Of note, the effect was more pronounced in ALT vs. TEP cells (Fig. 4B). Furthermore, miR-708 overexpressing U2OS (ALT) cells also showed decrease in CARF protein and was in line with the effect seen on its transcript level (Fig. 4C). Furthermore, stronger decrease in CARF protein in miR-708 overexpressing U2OS as compared to the MG63 (TEP) cells suggested that miR-708 inhibited CARF strongly in ALT cells. We next determined CARF expression level in isogenic ALT and TEP cell lines derived from a single fibroblast culture and found that the ALT cells, JFCF-6/T. 1L, JFCF-6/T.3C and JFCF-6/T.4D, have a lower level of CARF expression than their TEP counterparts, JFCF-6/T.6B and JFCF-6/T.G (Fig. 4D and Supplementary Fig. 2B). This was consistent with the higher level of miR-708 detected in ALT cells by array and qPCR assays. We next investigated the effect of CARF on the ALT-TMM using the C-circle assay. U2OS cells with stable high or low level of expression of CARF were subjected to C-circle assay. As shown in Fig. 4E, overexpression of CARF in ALT (U2OS) cells caused dose-dependent suppression of C-circles demonstrating that CARF negatively regulates ALT. It was consistent with the high level of miR-708 and corresponding low level of CARF in U2OS (ALT) cells. HeLa cells (with low or high level of exogenous CARF expression) used as control TEP cells did not show C-circles in either control or CARF-overexpressing derivatives.

### miR-708 regulates cell migration, invasion, and angiogenesis similarly in TEP and ALT cells

**Figure 5:**
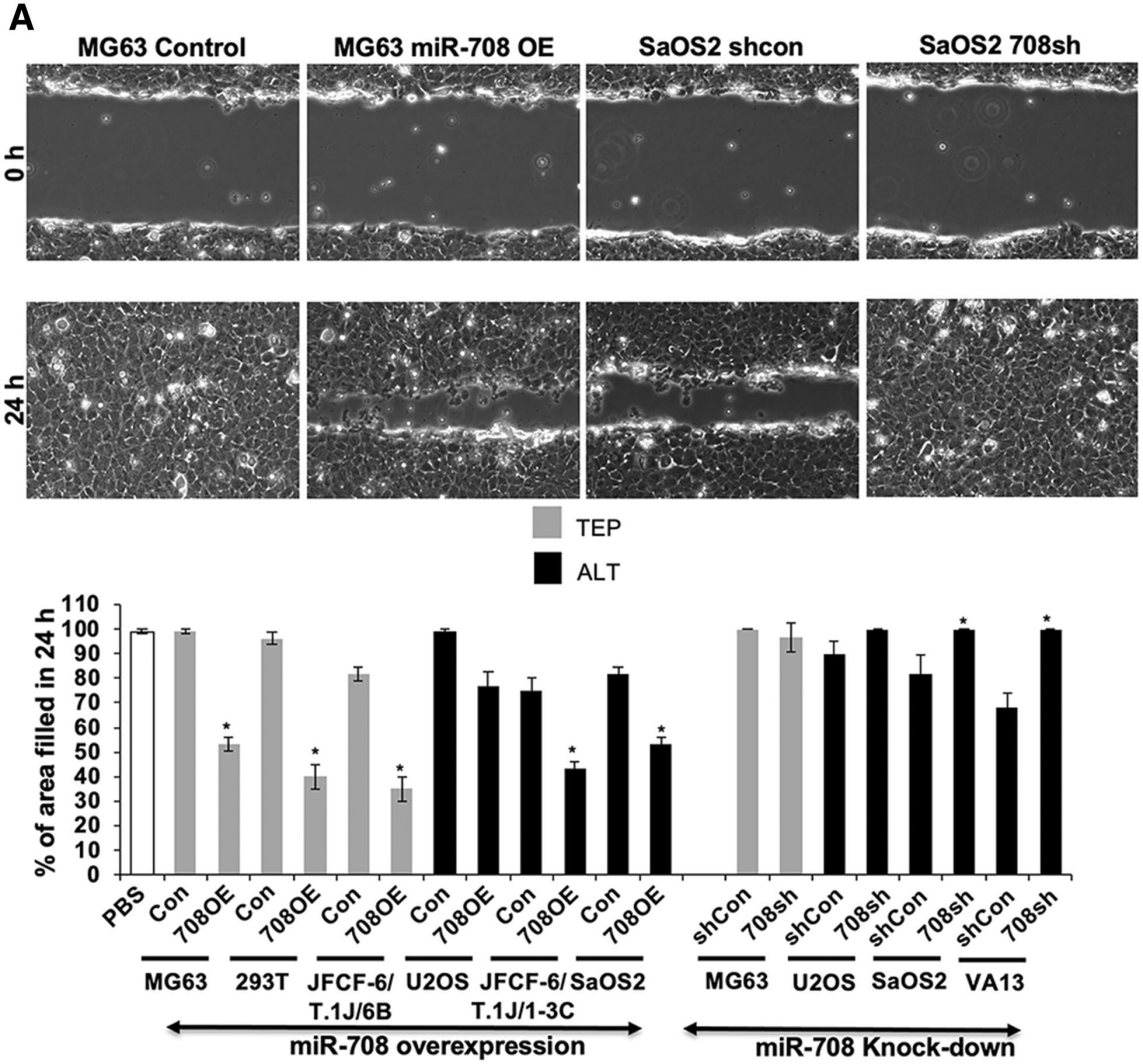

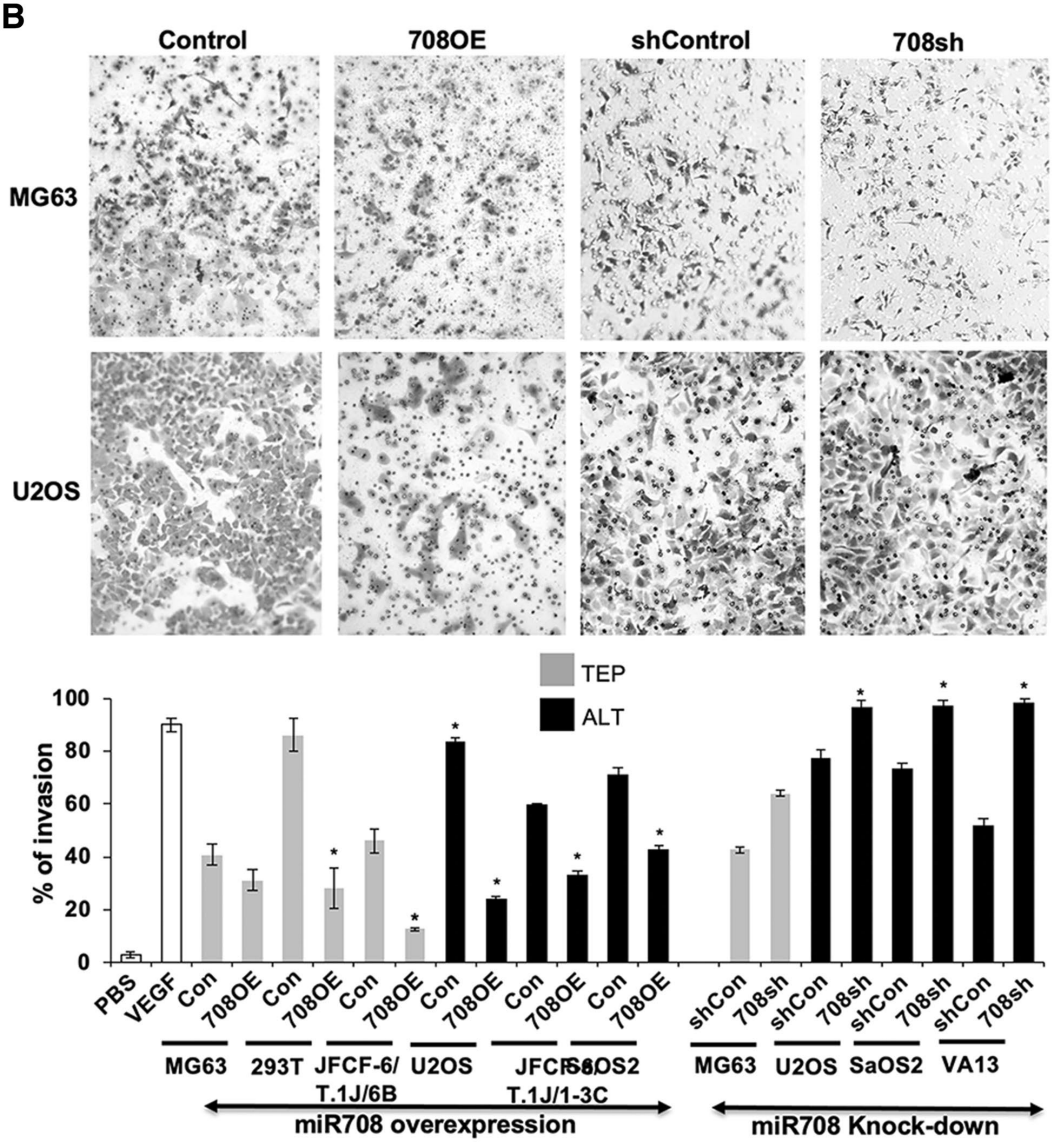

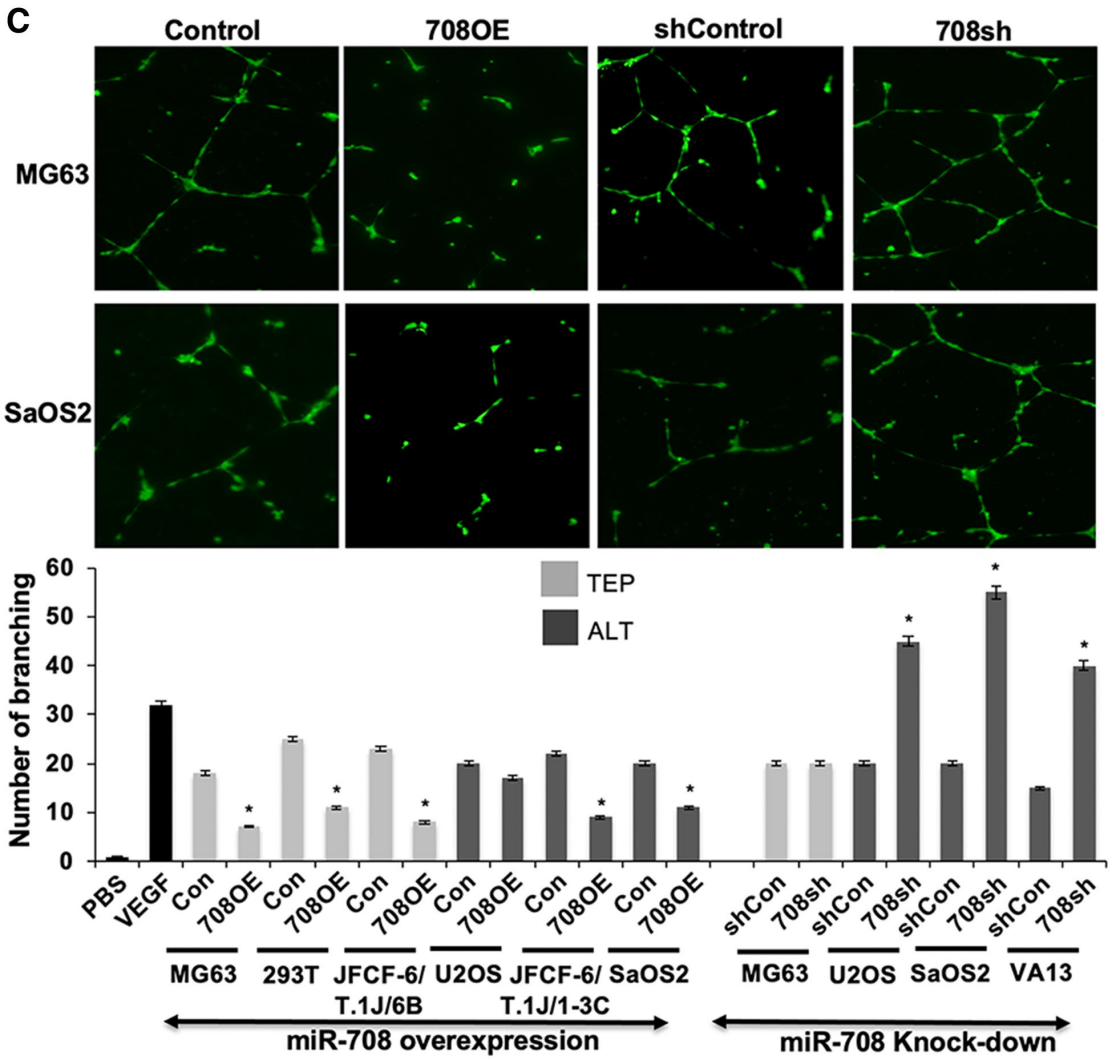
miR-708 has similar effects in migration, invasion, and angiogenesis in both TEP and ALT cells. (**A**) Wound healing migration assay. Top panels—representative images depicting Wound healing cell migration assay in miR-708-overexpressing MG63 (TEP) and miR-708-deficient SaOS2 (ALT) cells at 0 and 24 h after wound creation. Cells were plated into 6-well dishes and allowed to grow for 12 h, after which a scratch was created and cells were imaged immediately (0 h) and at 24 h. Quantitation of the cell migration assay (percentage of area filled at 24 h) in miR-708-overexpressing and -deficient TEP and ALT cell lines (mean ± SD, n = 3). Overexpression of miR-708 decreases migration similarly in both TEP and ALT cells. (**B**) Matrigel invasion assay. Top panels—representative images depicting the in vitro cell invasion assay in miR-708-overexpressing MG63 (TEP) and miR-708-deficient U2OS (ALT) cells. Invasion was induced by VEGF treatment and determined using Matrigel-coated membranes. Lower panel—quantitation of invasion assay in miR-708-overexpressing and -deficient TEP and ALT cell lines (mean ± SD, n = 3). Overexpression of miR-708 decreases invasion while its knock-down increases invasion similarly in both TEP and ALT cells. (**C**) Tube formation angiogenesis assay. Top panels—representative images depicting tube formation with conditioned media from miR-708-overexpressing MG63 (TEP) and miR-708-deficient SaOS2 (ALT) cells. Overexpression of miR-708 inhibits tube formation similarly in both TEP and ALT cell. PBS and VEGF (10 ng/mL) stimulated cells were used as negative and positive controls. Quantitation of tube formation (number of branches) in response to conditioned media from miR-708-overexpressing and -deficient TEP and ALT cell lines. Overexpression of miR-708 decreases angiogenesis similarly in both TEP and ALT cells. Each data set represents the mean ± SD for at least n = 3 independent experiments. Con: control for miR-708 expression; 708OE: miR-708 expression; shCon: control for miR-708 knock-down; 708sh: miR-708 knock-down using shRNA. *p < 0.05.

Based on the above data, we next performed functional analysis of miR-708 on cancer cell characteristics. Considering CARF as one of the targets of miR-708 and that it has recently been shown to regulate cell proliferation in a dose dependent manner, and play an important role in cancer cell migration, invasion and EMT transition^29–33^ we next determined the effect of miR-708 on these characteristics in TEP and ALT cells. We examined the effect of miR-708 on cell migration and invasion, using the scratch-wound healing assay. miR-708 overexpression inhibited cell migration to similar extents in both TEP and ALT cells (Fig. 5A). Whereas miR-708 compromised TEP (MG63) cells showed no effect, ALT cells showed a small to moderate increase in migration. We extended these findings to investigate the cell invasion capabilities of the miR-708-overexpressing and -deficient cells using the cell migration assay. As shown in Fig. 5B, miR-708 overexpression suppressed VEGF-induced cell invasion while miR-708 knock-down increased infiltration similarly in both TEP and ALT cells. These data suggested that miR-708 regulates cell migration and invasion. Its overexpression caused decrease in migration as well as invasion of TEP as well as ALT cells. On the other hand, miR-708 compromised cells showed increase in invasion. Of note, although U2OS cells possessed a low level of miR-708 expression, they showed altered migration and invasion characteristics upon its overexpression and knock-down. miR-708 OE U2OS cells showed small and insignificant decrease while knock-down cells showed increase in cell migration. On the other hand, invasion capacity of these cells was moderately affected by miR708 overexpression and knock-down (Fig. 5B). These data suggested that miR-708 has important role for cell migration and invasion characteristics of U2OS cells.

We next performed the tube formation assay for angiogenic capabilities using conditioned medium from the miR-708 overexpressing and deficient cell lines to stimulate branching of HUVEC cells. As shown in Fig. 5C, conditioned medium from miR-708 overexpressing cells caused a reduction in tube formation in both TEP and ALT cells. Although U2OS cells showed somewhat weak effect, it was suggestive that miR-708 may have an anti-angiogenic effect in most TEP and ALT cells. On the other hand, miR-708 suppression promoted tube formation only in ALT cells; miR-708 compromised TEP (MG63) cells did not show effect on tube formation. Taken together, these data suggested that miR-708 plays role in cell migration, invasion, and angiogenesis, especially in ALT cells that have evolved high expression of miR-708.

### miR-708 inhibited cell proliferation in TEP, but not ALT, cells

**Figure 6:**
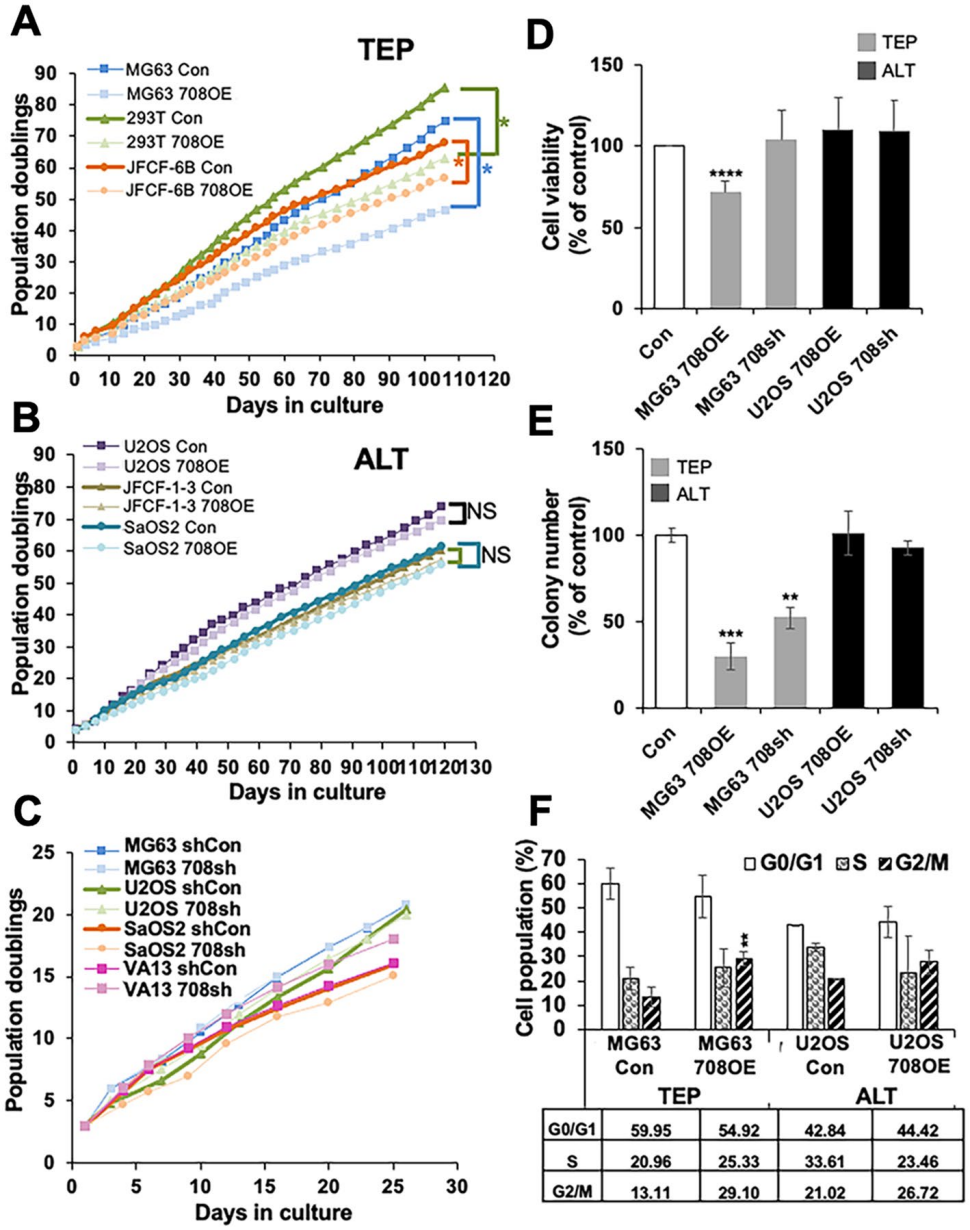
miR-708 regulates proliferation and colony formation only in TEP cells. (**A**–**C**) Growth curves of miR-708-overexpressing (**A**,**B**) and -deficient (**C**) TEP and ALT cell lines. (**A**) Overexpressing miR-708 decreased cell growth in TEP cells, (**B**) but not in ALT cells. (**C**) Depletion of miR-708 had no significant effect on growth rate of TEL (MG63) or ALT (U2OS, SaOS2, VA13) cells. (**D**) MTT assay in miR-708-overexpressing and -deficient TEP and ALT cell lines showing decrease in cell proliferation in miR-708-overexpressing TEP cells only (**E**) Colony forming assay showed that miR-708 overexpression decreased clonogenic capacity of TEP, but not ALT cells. (**F**) Cell cycle analysis of miR-overexpressing TEP and ALT cells showing strong G2/M arrest in TEP cells. Each data set represents the mean ± SD for at least 3 independent experiments. Con: control; 708OE: miR-708 expression; 708sh: miR-708 knock-down using short-hairpin interfering RNA. **p < 0.01; ***p < 0.001; ****p < 0.0001.

**Figure 7:**
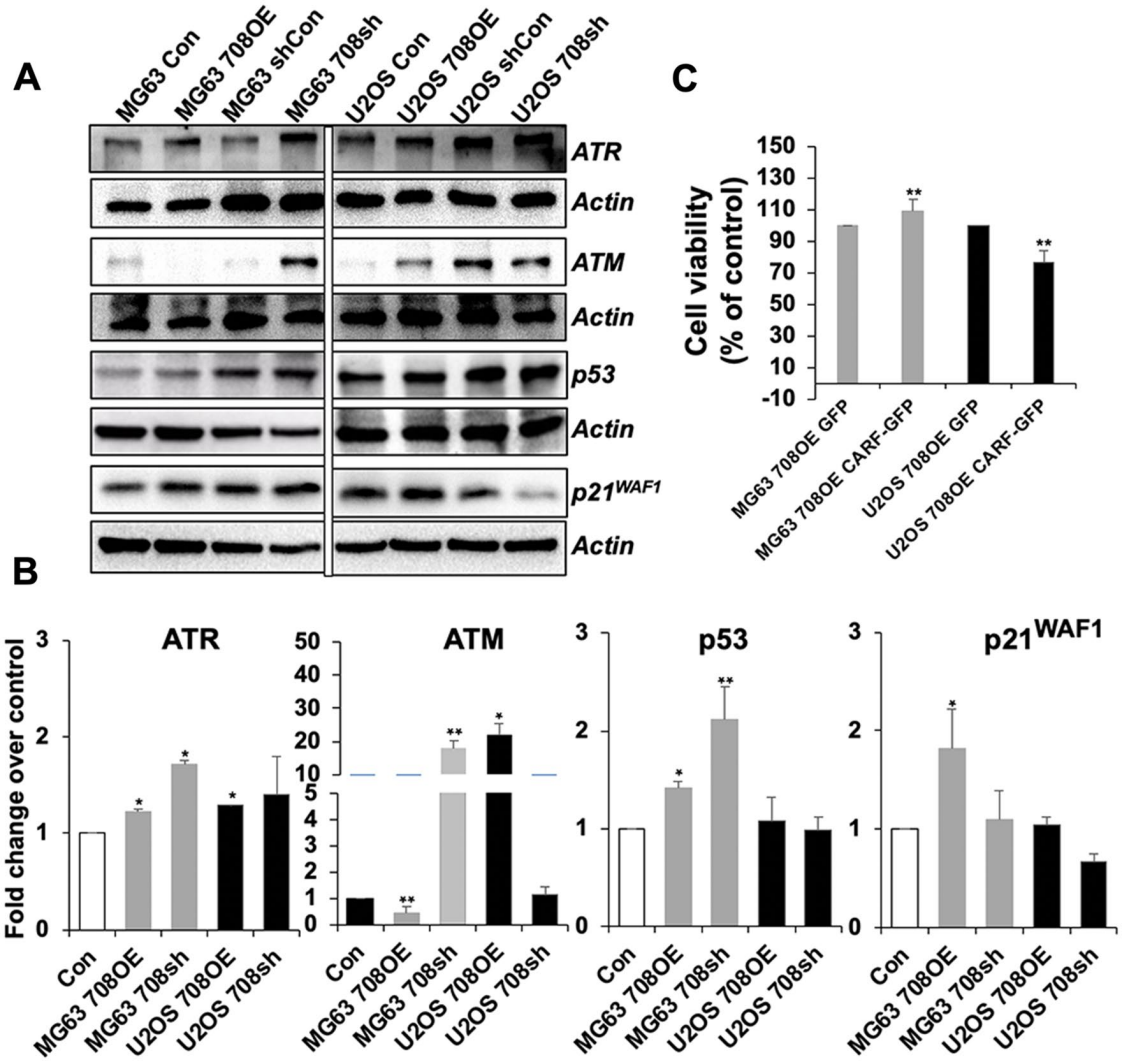
miR-708 differentially regulates CARF-ATM-p53-p21 signaling in TEP and ALT cells. (**A**) ATR, ATM, p53 and p21^WAF1^ protein levels. Whereas ATR did not show differential expression in miR-708-overexpressing and compromised cells, ATM expression was reduced and elevated by miR-708 overexpression and knock-down, respectively, only in TEP cells. ALT cells showed the opposite effect. Further, p53 was upregulated in TEP cells while it was unchanged in ALT cells. p21^WAF1^ was increased in miR-708-overexpressing TEP cells only and showed no change in ALT cells. (**B**) Quantitation of the data (mean ± SD) obtained from at least 4 independent Western blots. (**C**) CARF restoration in miR-708 overexpressing TEP and ALT cells. Whereas TEP cells showed an increase in cell growth in CARF restored miR-708 overexpressing cells, ALT cells showed a decrease. *p < 0.05; **p < 0.01**.**

Because CARF has been shown to act as a dual regulator of cell proliferation^31–35^, we next determined how miR-708 induced regulation of CARF controls cell proliferation in TEP and ALT cells. As shown in Fig. 6A, miR-708 overexpression led to a significant decrease in cell proliferation in TEP cells in long term cultures. This was consistent with the tumor suppressor function of miR-708^36,37^. In contrast, miR-708 overexpression in ALT cells did not have a detectable effect on cell proliferation rate (Fig. 6B). On the other hand, knockdown of miR-708 in either TEP or ALT cells did not cause any difference in their viability or colony forming efficacy (Fig. 6C). This was further confirmed by short term viability (MTT) assays that showed a decrease (~ 30%) in cell viability in miR-708 overexpressing TEP (MG63) cells as compared to the control and ALT cells that did not show any decrease (Fig. 6D). Long term colony-forming assay also showed a decrease in colony forming capacity in miR-708 overexpressing TEP, but not ALT, cells (Fig. 6E). Cell cycle analysis by flow cytometry showed that TEP cells with miR-708 overexpression were arrested in G2/M phase, whereas ALT cells did not show any change in cell cycle profile (Fig. 6F). These data suggested that inhibition of cell proliferation in miR-708 overexpressing TEP cells may predominantly be due to G2/M arrest that did not take place in ALT cells. In view of the reports that CARF knockdown causes G2/M arrest and apoptosis by regulation of DNA damage signaling^31^ and alterations in CARF level were previously linked to dysregulation of the ATR, ATM, and p53/p21^WAF1^ pathways, we explored the expression of these molecules in our miR-708-overexpressing and -deficient TEP and ALT cells. As shown in Fig. 7A,B, only a small and non-significant difference in ATR level was observed in miR-708 overexpression/knockdown derivatives of either the TEP or ALT cells. On the other hand, miR-708 overexpression caused decrease and its knock-down resulted in a remarkable increase in ATM protein in MG63 cells (Fig. 7A,B). Further, we observed an increase in p53 level in both miR-708-overexpressing and -deficient MG63 cells while its level was negligibly changed in the ALT cells. In contrast, p21^WAF1^ was consistently upregulated only in the miR-708-overexpressing MG63 (TEP) cells and remained unchanged in the miR-708-overexpressing U2OS (ALT) and other cells, which was consistent with the role of CARF as a transcriptional repressor of p21^WAF1^^33^. These differences in expression of CARF and p21^WAF1^ suggest that miR-708 may regulate DNA damage and growth arrest signaling differentially in TEP and ALT cells. Whereas targeting of CARF by miR708 yielded an increase in p21^WAF1^ in TEP cells, ALT cells showed no change in p21^WAF1^. Such changes may be responsible for the growth arrest in miR-708 overexpressing TEP, but not ALT. We next reconstituted CARF in miR-708 overexpressing TEP and ALT cells and found recovery from miR-708 overexpression induced growth arrest in TEP cells only (Fig. 7C). Taken together, these data suggested that miR-708 targets CARF and evokes differential regulation of p21^WAF1^-mediated cell proliferation in TEP and ALT cells.

## Discussion

ALT occurs in approximately 10–15% of tumors, many of which are biologically aggressive and currently difficult to treat, so to design new therapeutic modalities it is important to understand any intrinsic differences between cancers that use ALT and those that utilize TEP. Thus far, only a handful of studies have looked into the differences between ALT and TEP tumors. A comparative study between TEP and ALT cell lines was previously performed using a transcriptomic microarray, and it was found that 1305 genes were differentially expressed^37^. Interestingly, the ALT-TMM was demonstrated to be highly correlated to mesenchymal stem cell processes, which was supported by the fact that ALT is prevalent in sarcomas and astrocytomas^9^. Other studies have also shown differences in gene expression between TEP and ALT TMMs, and it was demonstrated that most ALT-associated genes belong to the DNA damage repair and recombination processes, including promyelocytic leukemia (PML), RAD50 double strand break repair protein, RAD51 recombinase, RAD52, structural maintenance of chromosomes 5 (SMC5), structural maintenance of chromosomes 6 (SMC6), Bloom syndrome RecQ-like helicase (BLM), breast cancer type 1 (BRCA1), DNA topoisomerase III alpha (TOP3A), RecQ-mediated genome instability 1 (RMI1), MMS21 SUMO ligase, MRE11A double strand break repair nuclease homolog A, Nibrin (NBS1), Flap structure-specific endonuclease 1 (FEN1), MUS81 structure-specific endonuclease subunit, FA complementation group D2 (FANCD2), and FA complementation group A (FANCA), as well as the Shelterin proteins, telomeric repeat binding factor (TRF) 1 and TRF2^35–38^. Another study showed that synaptonemal complex central element protein 1 (SYCE1), NBS1, PML, NEDD4‐binding protein 2 (N4BP2), sarcoma antigen 1 (SAGE1), growth arrest‐specific protein 8 (GAS8), and structural maintenance of chromosomes protein 1β (SMC1β) are also specifically associated with ALT cells^39^. Many of these genes appear to be involved in the formation and maintenance of APBs^40^. Moreover, it has been identified that the ATP-dependent helicase/X-linked helicase II (ATRX), a chromatin remodeler, and its partner DAXX (death domain associated protein) are repressors of ALT TMM^41^ and that loss of one or both of these proteins induces telomere dysfunction that leads to DNA damage and repair responses as well as cell cycle checkpoint activation^42^. ALT has also been characterized by elevated levels of telomeric DNA damage and repair^43^. Although these studies provide many new potential targets to investigate to differentiate between TEP and ALT tumors, differences in their regulatory mechanisms are still largely unknown. A previous transcriptomic study by Lafferty-Whyte et al.^44^ aimed to distinguish TEP and ALT cells by gene expression signatures but was unable to determine the mechanisms governing the differential gene expression. Therefore, in this study, we set out to compare the differential expression of miRNAs between isogenic TEP and ALT cells. miRNAs have been shown to have wide-ranging effects on gene expression: most miRNAs target multiple transcripts and regulate multiple signaling cascades. We found that miR-708 was consistently expressed at a higher level in ALT cell lines and tumors than in TEP and C-circle-negative cell lines, cancers and normal tissue (Fig. 1), which suggested that it may have a differential function in these cell types.

Using four online target prediction programs to determine potential targets of miR-708, we identified MRE11A and BRCA1, major proteins that function in DNA damage response and repair and have consistently been demonstrated to be essential for the ALT-TMM^41^. Of note, none of the target analysis programs revealed any other targets involved directly in regulation of telomere/telomerase structure or function. We found that MRE11A and BRCA1 are both directly targeted by miR-708 at their 3′ UTR, although only BRCA1 showed differential regulation at the protein level, suggesting that MRE11A levels could be maintained despite the decrease in transcripts by post-translational mechanisms, such as an increase in protein stability. However, their functional roles in TEP and ALT cells may be different, and hence warrant further investigation.

CARF was also identified as a potential target of miR-708 by the target prediction programs. It has previously been shown to play a crucial role in DNA damage response and to be a major cell fate regulator in tumor cells through its role as a regulator of p53 and p21^WAF1^ activities, whereby its suppression leads to apoptosis, its moderate upregulation results in growth arrest, and its excessive increase induces increased proliferation and malignant transformation^31–34^. Thus, we predicted that CARF may be a link for several different processes that may induce and regulate ALT. miR-708 overexpressing and depleted cells showed a decrease and increase in CARF, respectively, both in TEP and ALT cells (Fig. 4C). Downstream effectors of CARF, however, were differentially regulated in these cells. No differences were observed in ATR subsequent to miR-708 overexpression (CARF decrease) and knockdown (CARF increase) (Fig. 7A). Although replication stress and dysfunctional DNA repair regulated by ATR are major features of ALT, a previous study also showed that TEP and ALT cells do not show difference with regard to sensitivity to ATR inhibitors, suggesting that ATR may affect cell viability similarly between TEP and ALT cells^27,40,45^. In contrast, we observed that miR-708 overexpression caused an increase in ATM in ALT cells (Fig. 7A), in line with previous findings showing that CARF downregulation increases ATM expression and activity^31^, while it was dramatically reduced and increased in miR-708-overexpressing and -deficient TEP cells, respectively. ATM is generally activated only in response to double-stranded breaks in TEP cells, but it is found to be constitutively active in APBs of ALT cells^42^ where it is postulated to facilitate DNA repair^46,47^. However, it is not known whether ATM has any differential function in the two TMM cell types.

Our results further demonstrated that despite the differential gene expression of ATR and ATM in the ALT cells, expression levels of p53 and p21^WAF1^ were unchanged in the miR-708-overexpressing ALT (U2OS) cells, whereas they were both increased in the miR-708-overexpressing TEP cells, in line with the observed decrease in cell viability of these cells (Fig. 7). Interestingly, mutant p53 has been implicated as a contributing factor in inducing ALT and maintaining its status, which is supported by the observation that an overwhelming majority of ALT tumors have a p53 mutation^14^, and it is suggested that mutant p53 regulates the formation of APBs via p21^WAF1^ and HP1 while suppressing growth arrest^48,49^. A recent study suggested that ALT tumors have evolved to withstand the apoptosis-promoting effects of p53 at low levels, and that a higher level of p53 is required to induce apoptosis in these tumors^50^. Total p53 level was unchanged in the miR-708-modified U2OS cells, which harbor wild type p53, despite the changes in CARF expression, corroborating the lack of change in cell viability in these cells. In contrast, p53 level was increased in both miR-708-overexpressing and -deficient MG63 cells, such that observed growth arrest signaling from this molecule was not likely possible. However, it was previously shown that CARF can directly regulate p21^WAF1^, the downstream effector of p53, to induce cell growth arrest and/or apoptosis, especially in a p53-null background^33,51^. Herein, we found that p21^WAF1^ was increased in the miR-708-overexpressing, but not in the miR-708-deficient MG63 cells, which was in line with the decrease in cell viability and growth arrest observed in these cells. Thus, these findings clearly show that CARF signaling is differentially regulated by miR-708 in TEP and ALT cells, and miR-708-mediated repression of CARF increased p21^WAF1^ activity, which ultimately led to cell cycle arrest at G2/M and suppression of cell growth only in TEP cells. This is supported by a recent study that found that another miRNA, miR-335, also targets CARF led to upregulation of p21^WAF1^ and induced cell cycle arrest^28^. On the other hand, ALT cells have higher endogenous levels of miR708 and low level of CARF expression, which suggests that ALT cells may have evolved a mechanism to escape miR708/CARF/p21^WAF1^-mediated suppression of cell proliferation that is seen in TEP cells.

Regulation of various carcinogenic processes, including cell proliferation, migration, invasion, and angiogenesis by miR-708 was also compared between TEP and ALT cells using overexpression and knock-down models. It was demonstrated that miR-708 suppressed migration, invasion, and angiogenesis to a similar degree in both TEP and ALT cells (Fig. 5), while it reduced cell proliferation predominantly in TEP cells as compared to ALT cells, at least in part through differential regulation of the CARF pathway, including its associated proteins, ATM, ATR, and ERK (Fig. 7A,B)^30–33^. Thus, intrinsic differences between TEP and ALT cells exist with regard to regulation of cell growth by miR-708 which contribute to the underlying mechanistic variances beyond those governed by the TMMs that are observed between these two tumor types. In order to further confirm the role of CARF, we performed CARF restoration in miR-708 overexpressing TEP and ALT cells. As shown in Fig. 7C, miR-708 induced growth arrest in TEP cells was neutralized by CARF restoration. On the other hand, ALT cells showed a decrease in cell viability, indicating differential regulation of cell proliferation by miR-708 and its target CARF in TEP and ALT cells.

**Figure 8:**
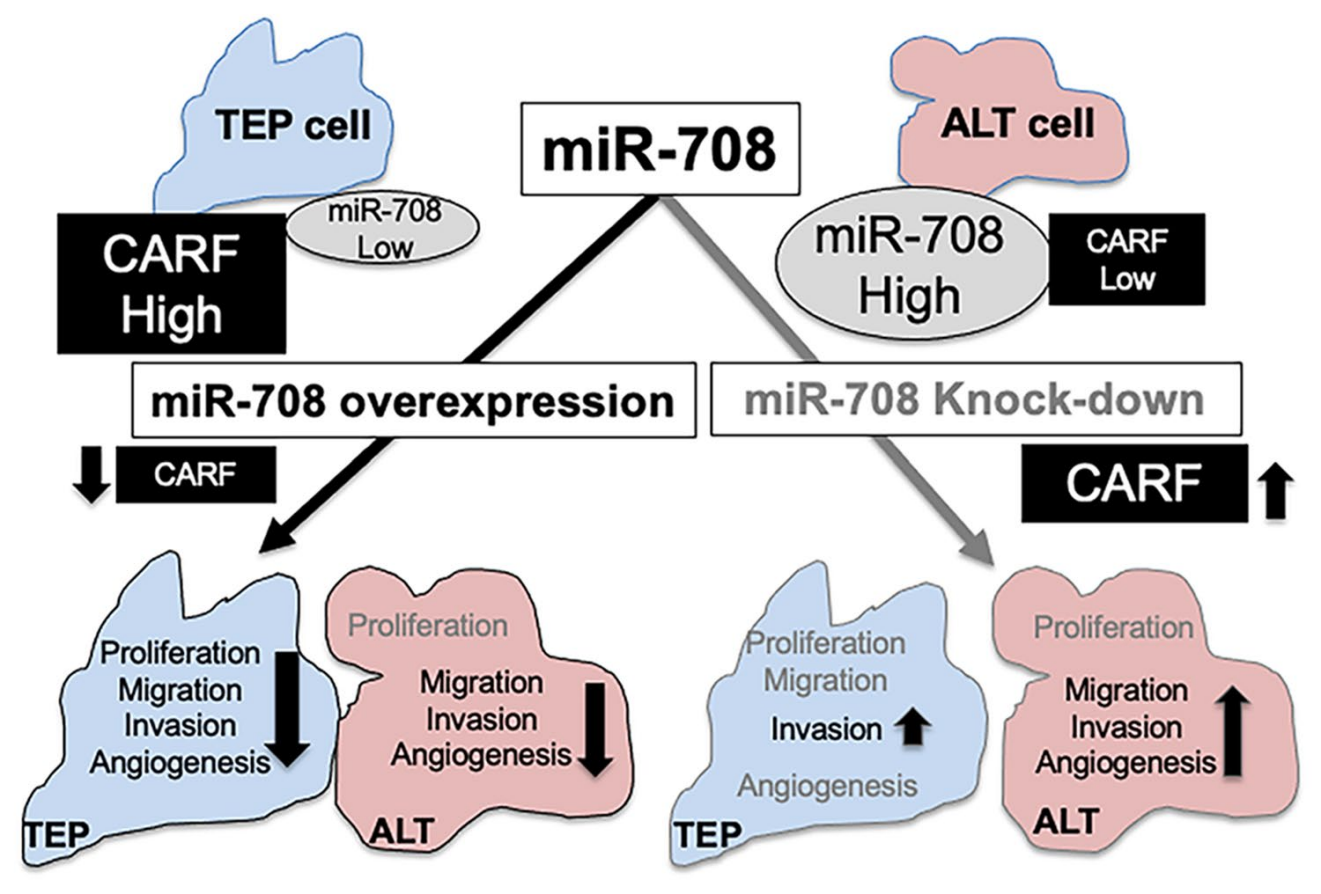
Schematic presentation of differential expression of miR-708 and its target CARF that evokes differential regulation of cell proliferation in TEP and ALT cells.

Our results led us to propose that ALT cells, which harbor a high level of endogenous miR-708 and low level of CARF protein, have evolved to evade the effect of miR-708 upregulation on cell proliferation (Fig. 8). On the other hand, TEP cells possess a lower level of endogenous miR-708 and are sensitive to its modulation; overexpression of miR-708 and subsequent downregulation of CARF caused growth arrest and inhibition of malignant characteristics as supported by molecular data that was in line with CARF-p21^WAF1^ axis signaling reported previously^31–35,52,53^. CARF shows genomic amplification and enrichment in a variety of tumors and has been shown to play an important role in EMT signaling^35^, so induction of miR-708 overexpression may provide a therapeutic approach for TEP tumors that have low level of endogenous expression of miR-708. On the other hand, and in sharp contrast, ALT cells with high level of endogenous miR-708 were refractory to the effect of miR-708 overexpression on their proliferation, although migration and invasion were affected similarly to TEP cells. Of note, the knock-down of miR-708 caused activation of malignant (cell migration, invasion and tube formation) characteristics (Fig. 7) in ALT cells; TEP cells showed a lesser effect of miR-708 silencing, such that only invasion was increased in these cells. ALT activity has been shown to be associated with elevated levels of telomeric DNA damage and repair, an essential feature of TMM. At the same time, excessive levels of telomeric DNA repair are highly deleterious to ALT cells^54^. In these premises, downregulation of DNA repair proteins by overexpression of miR-708 in ALT cells may serve to prevent excessively elevated levels of DNA repair.

In summary, our study is the first to (i) compare the miRNA profile between TEP and ALT cells, (ii) to identify miR-708 as highly expressed in ALT cells and (iii) to identify miR-708 as a deregulator of CARF-p21 ^WAF1^ signaling and DNA damage response in TEP cells. We demonstrate that miR-708 is a tumor suppressor miR that operates differently in TEP and ALT cells. Furthermore, we identified CARF as a target of miR-708 that mediates, at least in part, differential tumor suppressor activity of miR-708 in TEP and ALT cells. Such information is extremely important for therapeutic choices, outcomes and prognosis in treatment of TEP and ALT tumors.

## Supplementary Information

Supplementary Figures.

## Data Availability

The datasets/supporting data obtained and analyzed during the current study will be available from the corresponding authors through request.

## Abbreviations

TMM: Telomere length maintenance mechanism
ALT: Alternative lengthening of telomeres
TEP: Telomerase-positive
APBs: ALT-associated promyelocytic leukemia protein nuclear bodies
miRNAs: MicroRNAs
TRAP: Telomeric repeat amplification protocol
ELISA: Enzyme-linked immunosorbent assay
HUVECs: Human umbilical vessel endothelial cells
PVDF: Polyvinylidene fluoride
ECL: Enhanced chemiluminescence
OE: Overexpressing
EMT: Epithelial-mesenchymal transition
SMC5: Structural maintenance of chromosomes 5
SMC6: Structural maintenance of chromosomes 6
BLM: Bloom syndrome RecQ-like helicase
BRCA1: Breast cancer type 1
RMI1: RecQ-mediated genome instability 1
FEN1: Flap structure-specific endonuclease 1
FANCD2FA: Complementation group D2
FANCA: FA complementation group A
TRF: Telomeric repeat binding factor
SYCE1: Synaptonemal complex central element protein 1
N4BP2: NEDD4‐binding protein 2
SAGE1: Sarcoma antigen 1
GAS8: Growth arrest‐specific protein 8
SMC1β: Structural maintenance of chromosomes protein 1β
ATRX: ATP-dependent helicase/X-linked helicase II
DAXX: Death domain associated protein
