# Supplementary material for: Functional characterization of miR-708 microRNA in telomerase positive and negative human cancer cells
Source: Sci Rep. 2021 Aug 23;11:17052. doi: 10.1038/s41598-021-96096-y (PMC8382839; doi:10.1038/s41598-021-96096-y)
Supplement: Supplementary file 1 — Supplementary Figures. [file 41598_2021_96096_MOESM1_ESM.pdf]

Functional characterization of miR-708 microRNA in telomerase positive and negative human cancer cells

Zeenia Kaul[1,2,3], Caroline TY Cheung[2], Priyanshu Bhargava[2], Anissa Notifa Sari[2], Yue Yu[2], He Huifu[2], Hemant Bid[5], Jeremy D Henson[6], Joanna Groden[3,4], Roger R. Reddel[1], Sunil C. Kaul[2*] & Renu Wadhwa[2*]

Supplementary Information
&
FULL BLOTS

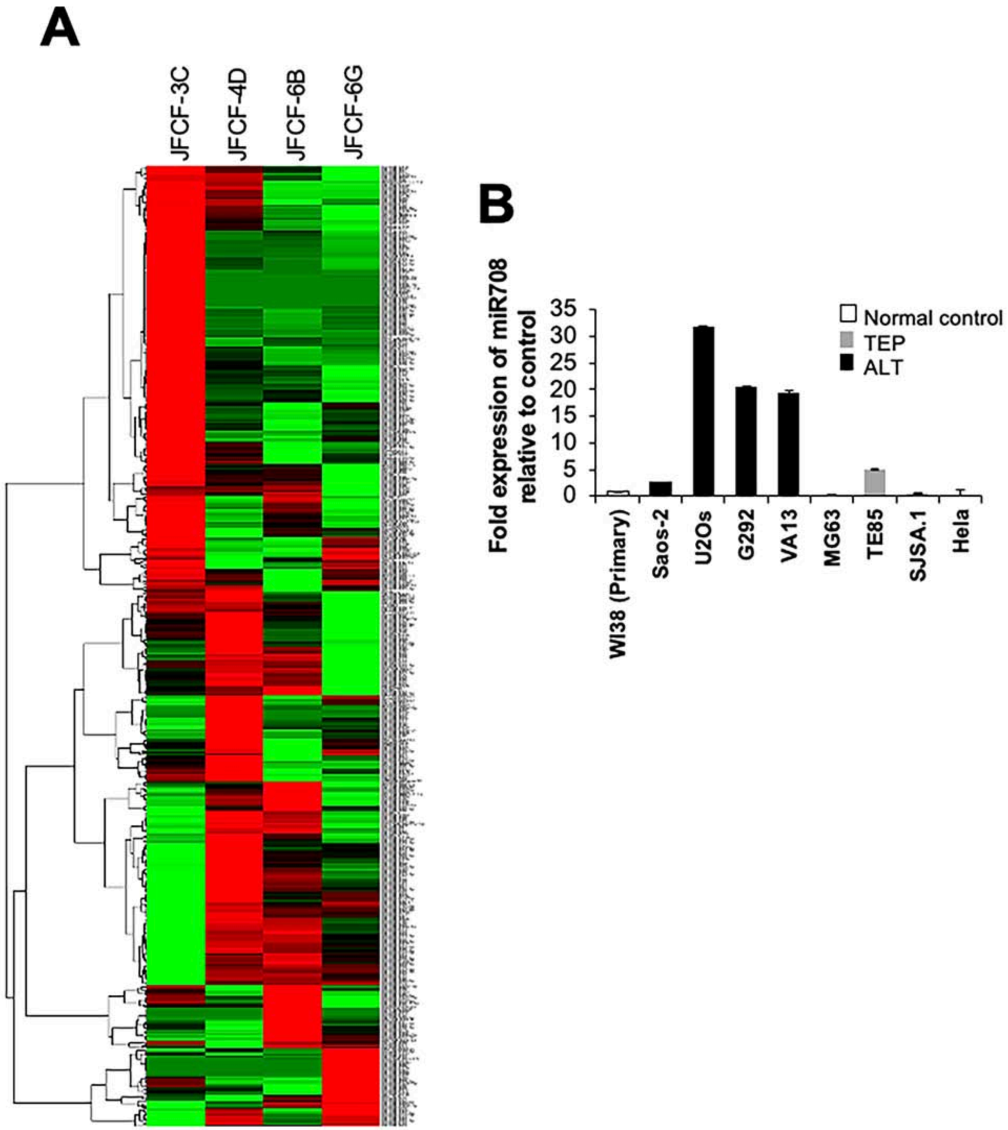

Supplementary Figure 1. (A) Heatmap of the microarray analysis. Of the 817 microRNA species tested on the microarray, 515 genes were found to be differentially expressed in at least one of the cell lines and were thus depicted in this supervised clustering heat map. Also, around 300 genes were either not expressed, or not differentially expressed amongst all TEP and ALT cells. The microarray genes were median-centered and clustered by average linkage. As seen from the heatmap, there were not that many miRNA genes that were consistently expressed in a similar manner in ALT versus TEP cells. (B) Comparison of miR-708 levels between various TEP and ALT cells by qPCR. miRNA expression is shown as fold expression as compared to control, normal (WI38) cells. miR708, was consistently downregulated in TEP cells and upregulated in ALT cells.

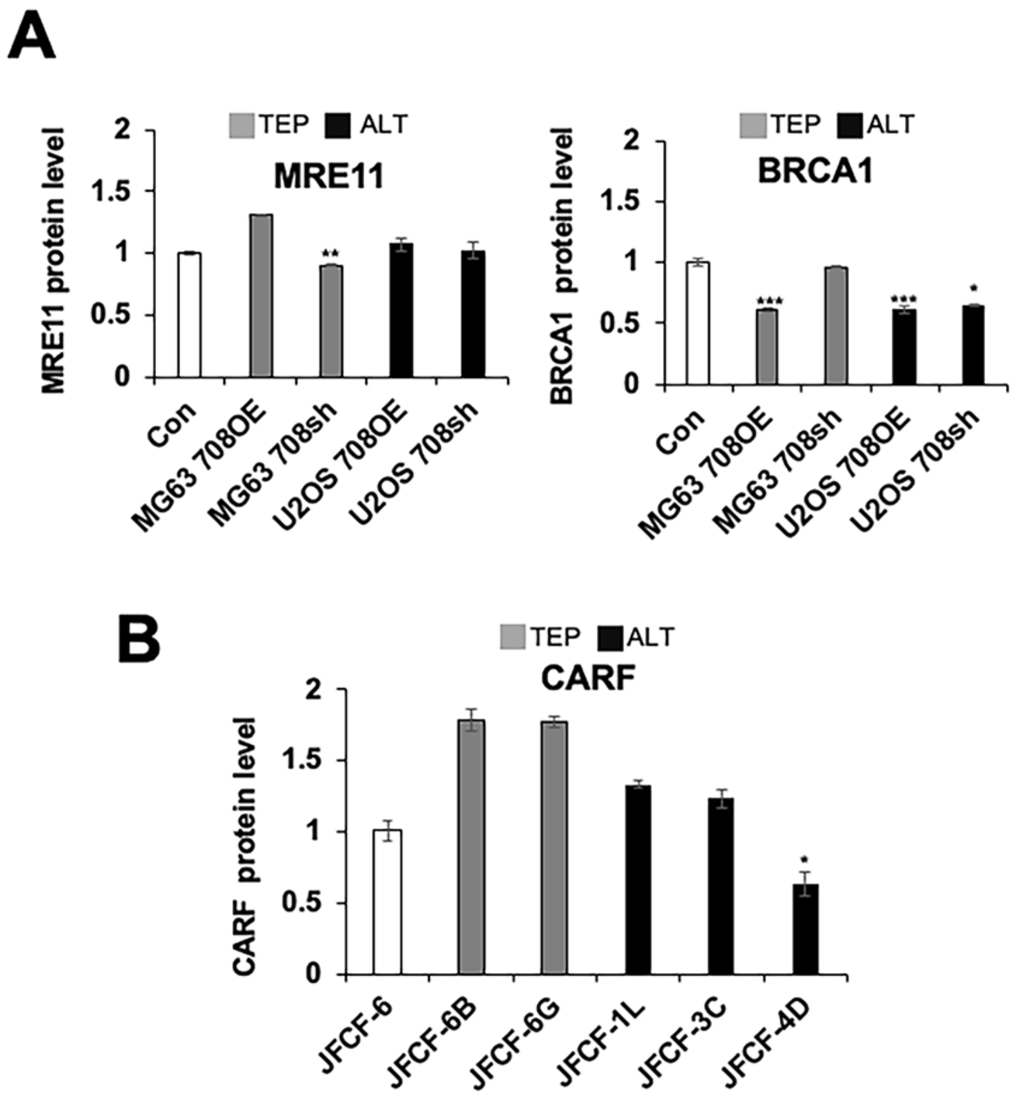

Supplementary Figure 2. (A) Quantitation of the Western blotting data shown in Figure 3E.
(B) Quantitation of the Western blotting data shown in Figure 4D.
*p<0.05; **p<0.01; ***p<0.001.

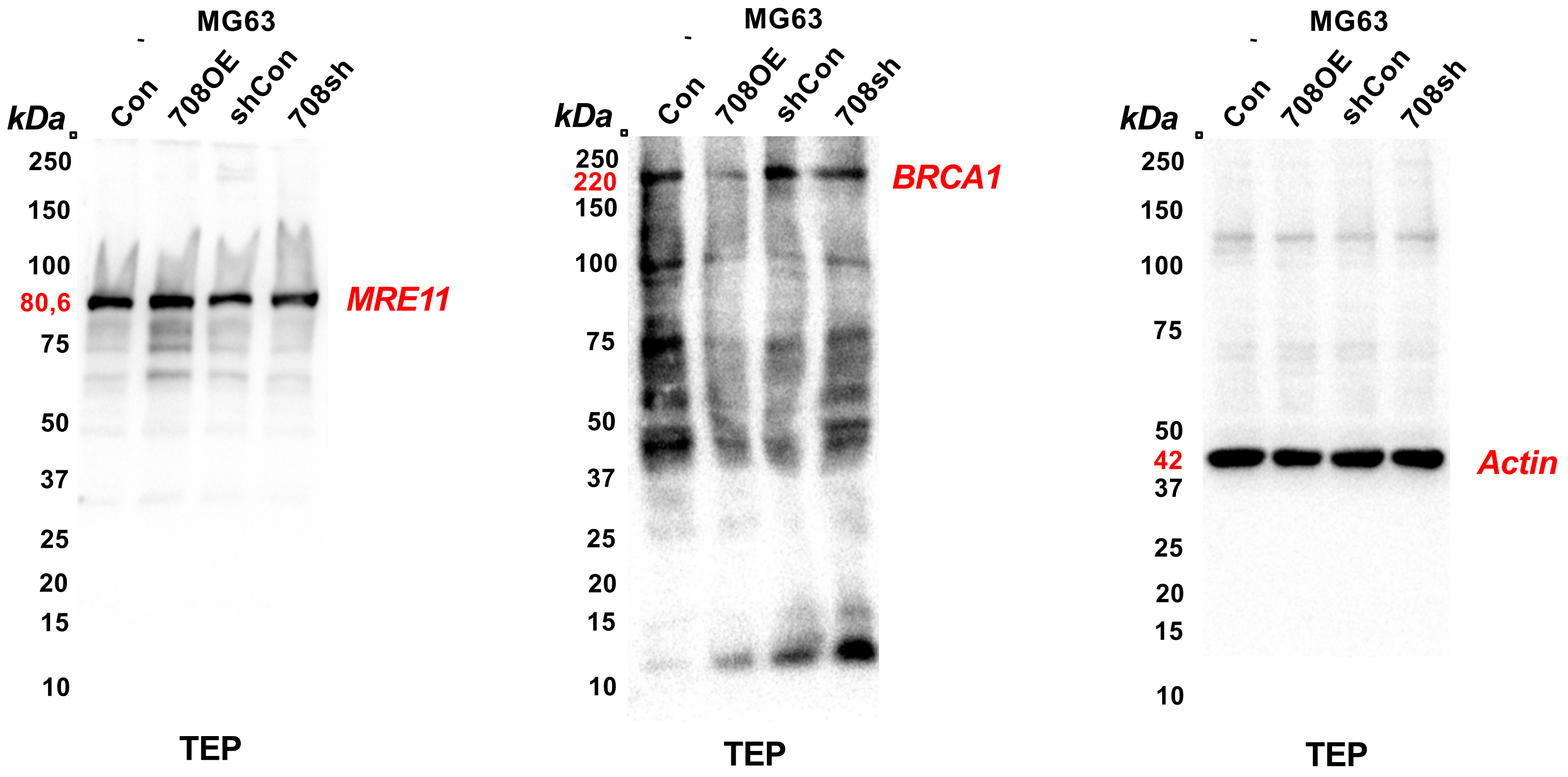

Western blot of proteins of interest (MRE11 and BRCA1) and respective actin expression presented in Figure 3E

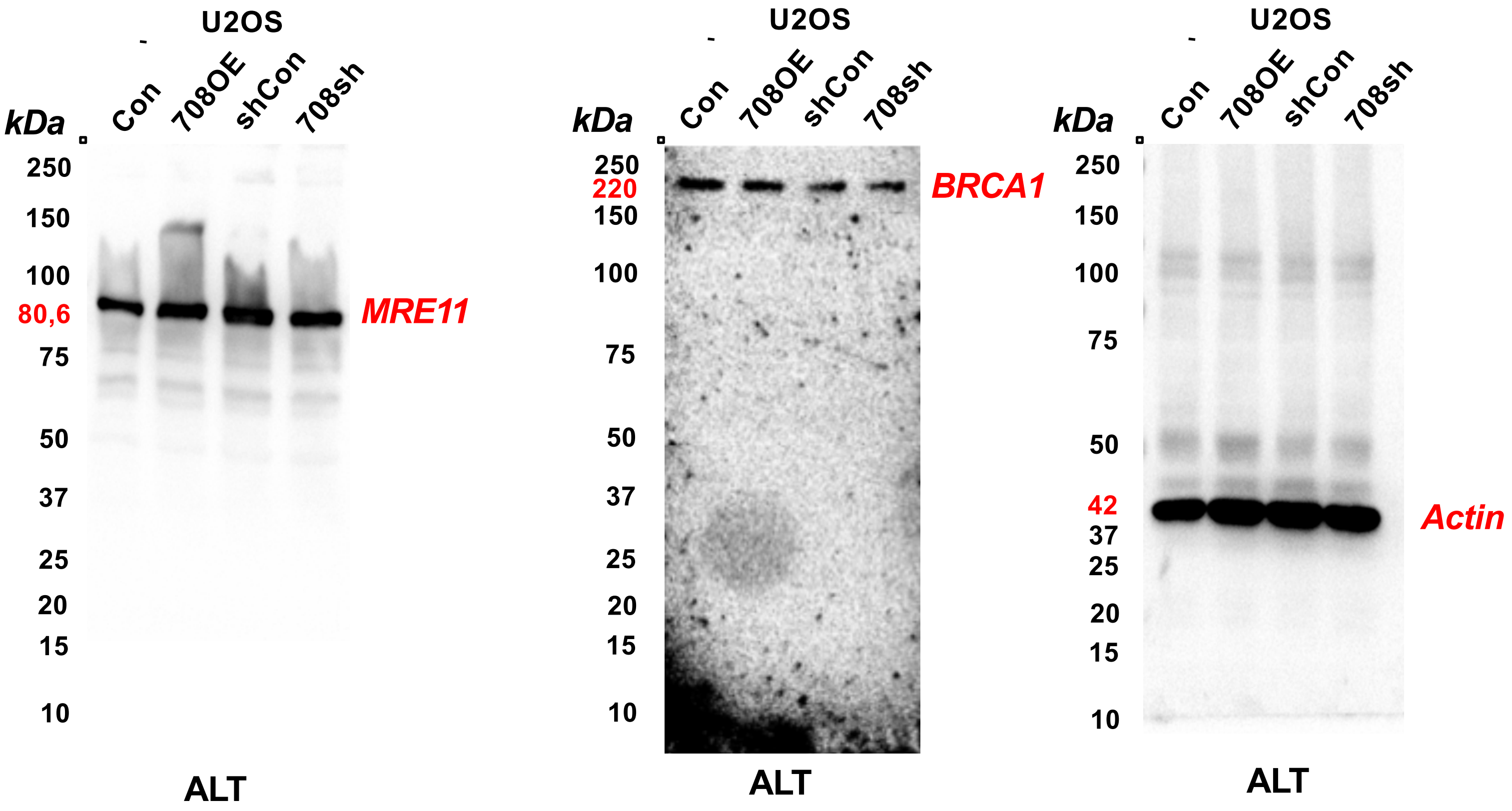

Western blot of proteins of interest (MRE11 and BRCA1) and respective actin expression presented in Figure 3E

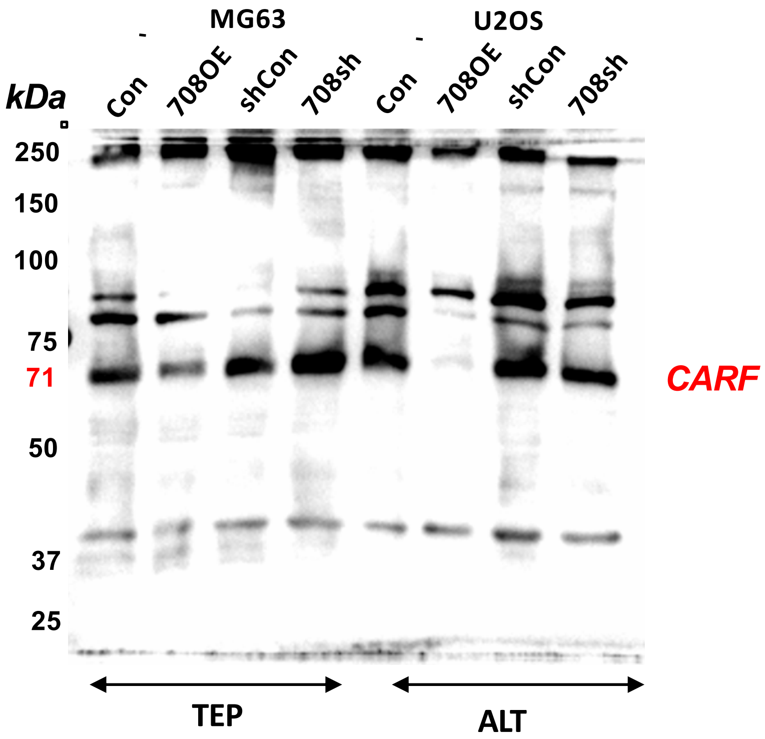

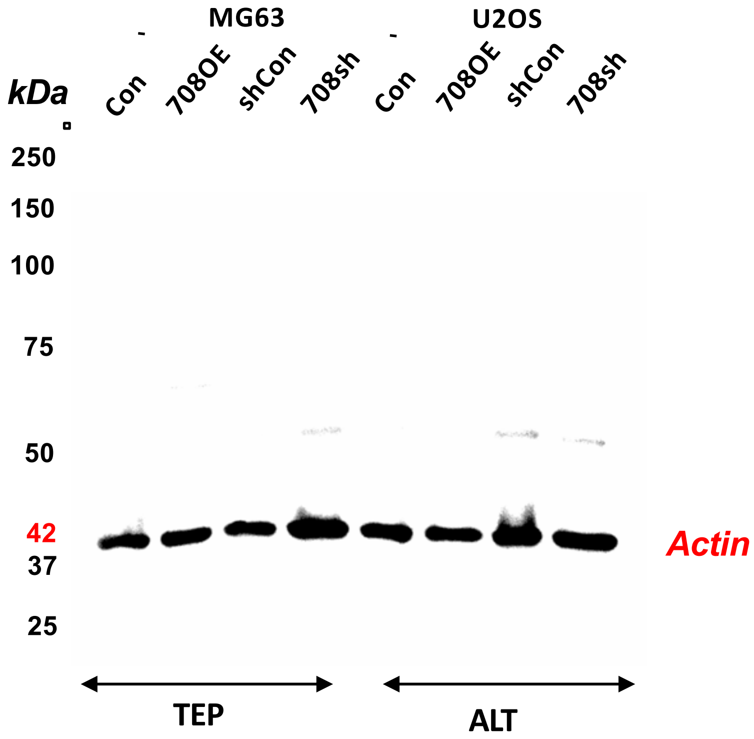

Western blot of protein of interest (CARF) and respective actin expression presented in Figure 4C

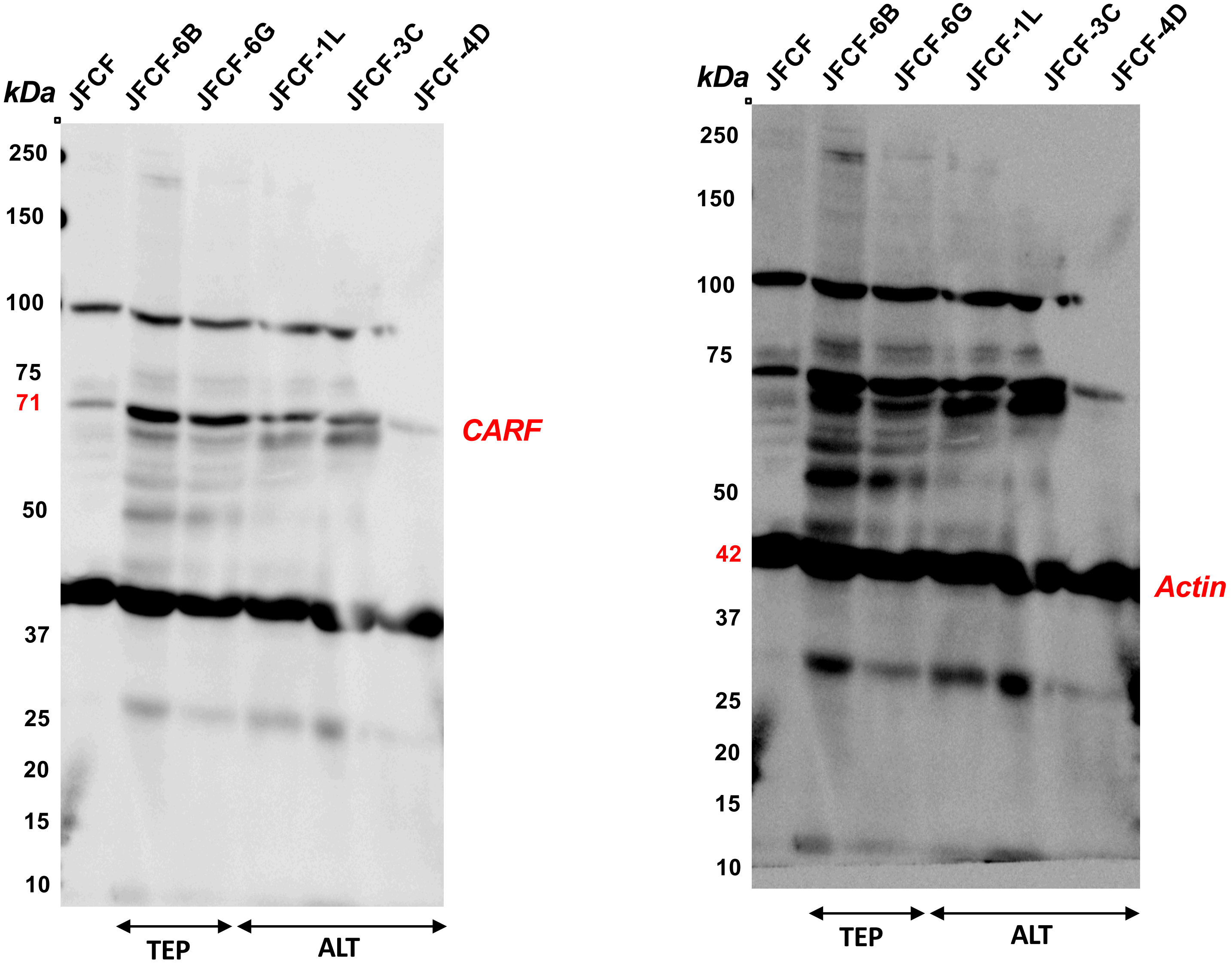

Western blot of protein of interest (CARF) and respective actin expression presented in Figure 4D

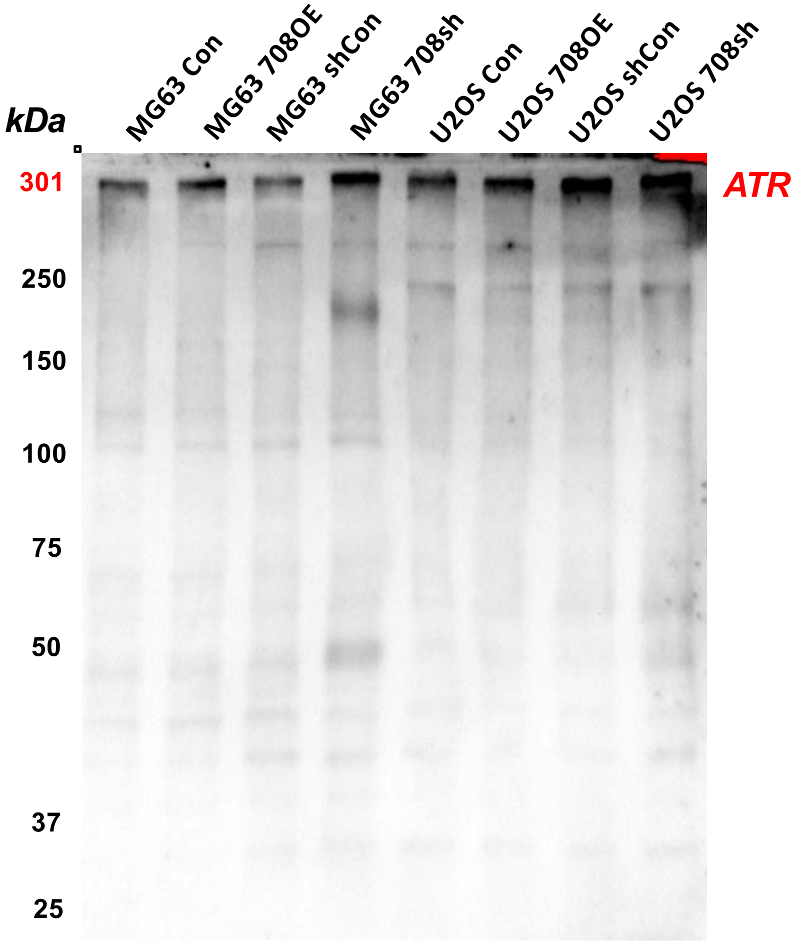

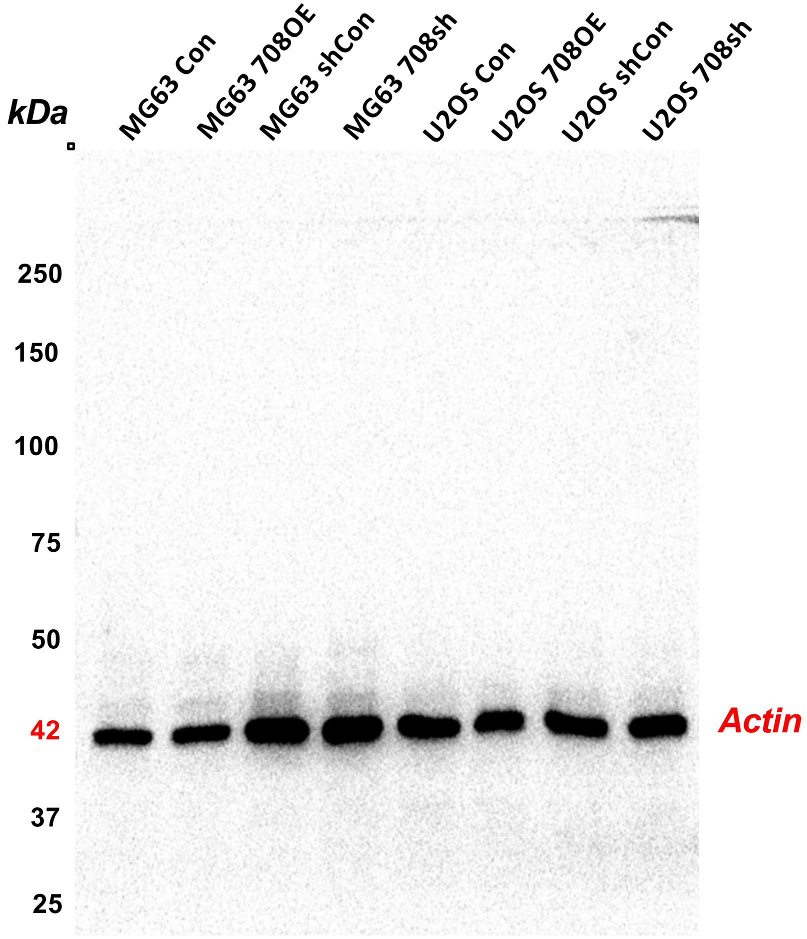

Western blot of protein of interest (ATR) and respective actin expression presented in Figure 7A

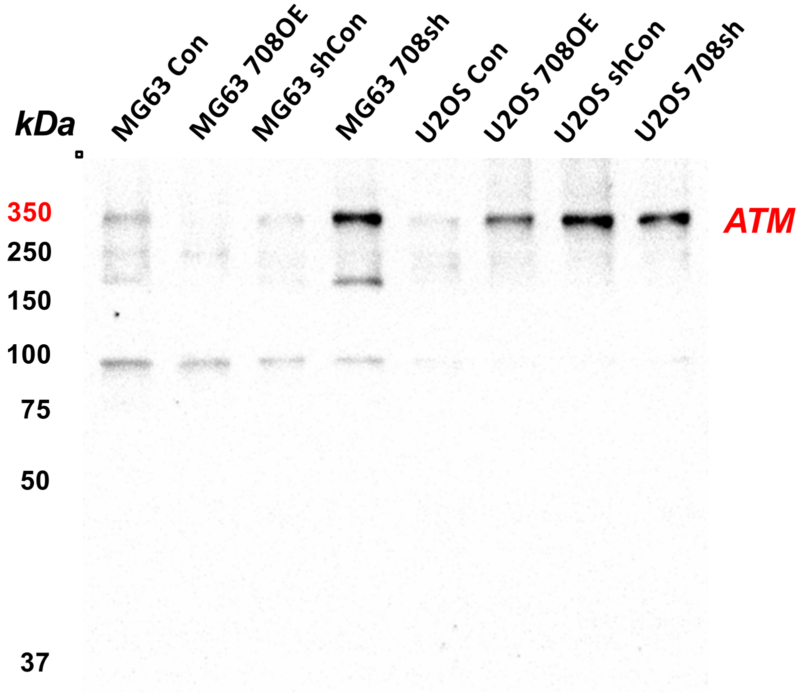

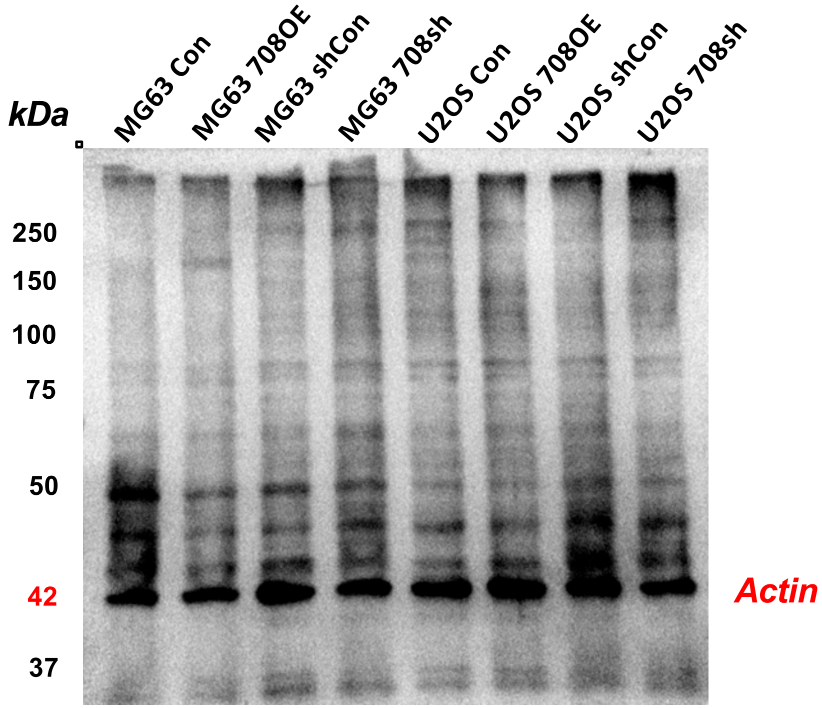

Western blot of protein of interest (ATM) and respective actin expression presented in Figure 7A

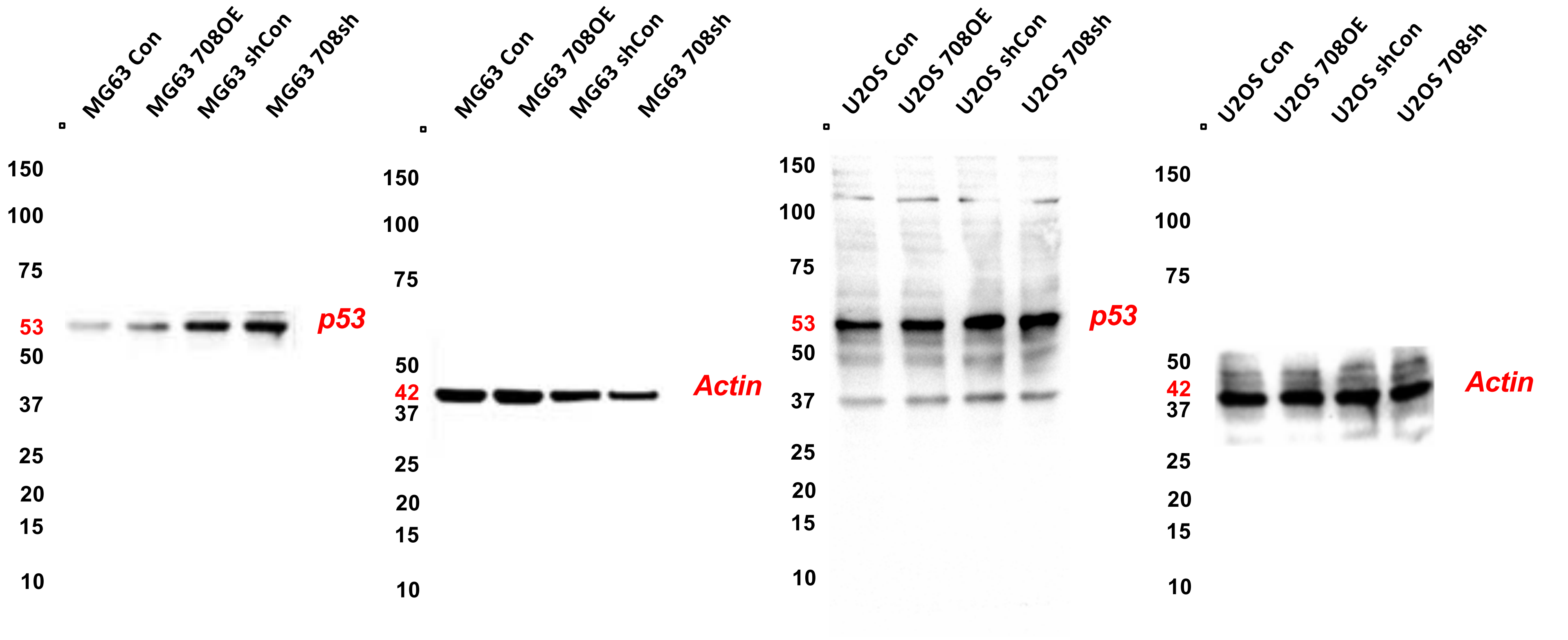

Western blot of protein of interest (p53) and respective actin expression presented in Figure 7A

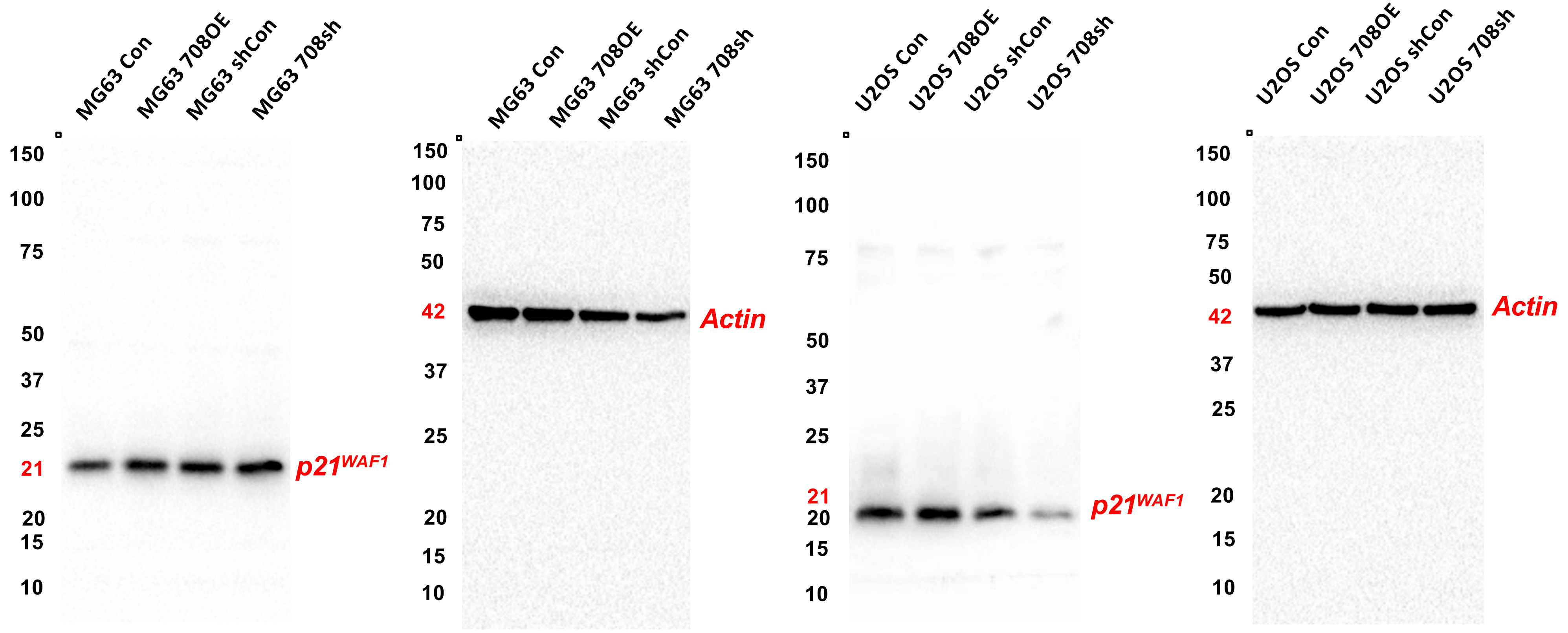

Western blots of protein of interest (*p21*^{WAF1}) and respective actin expression as given in Figure 7A

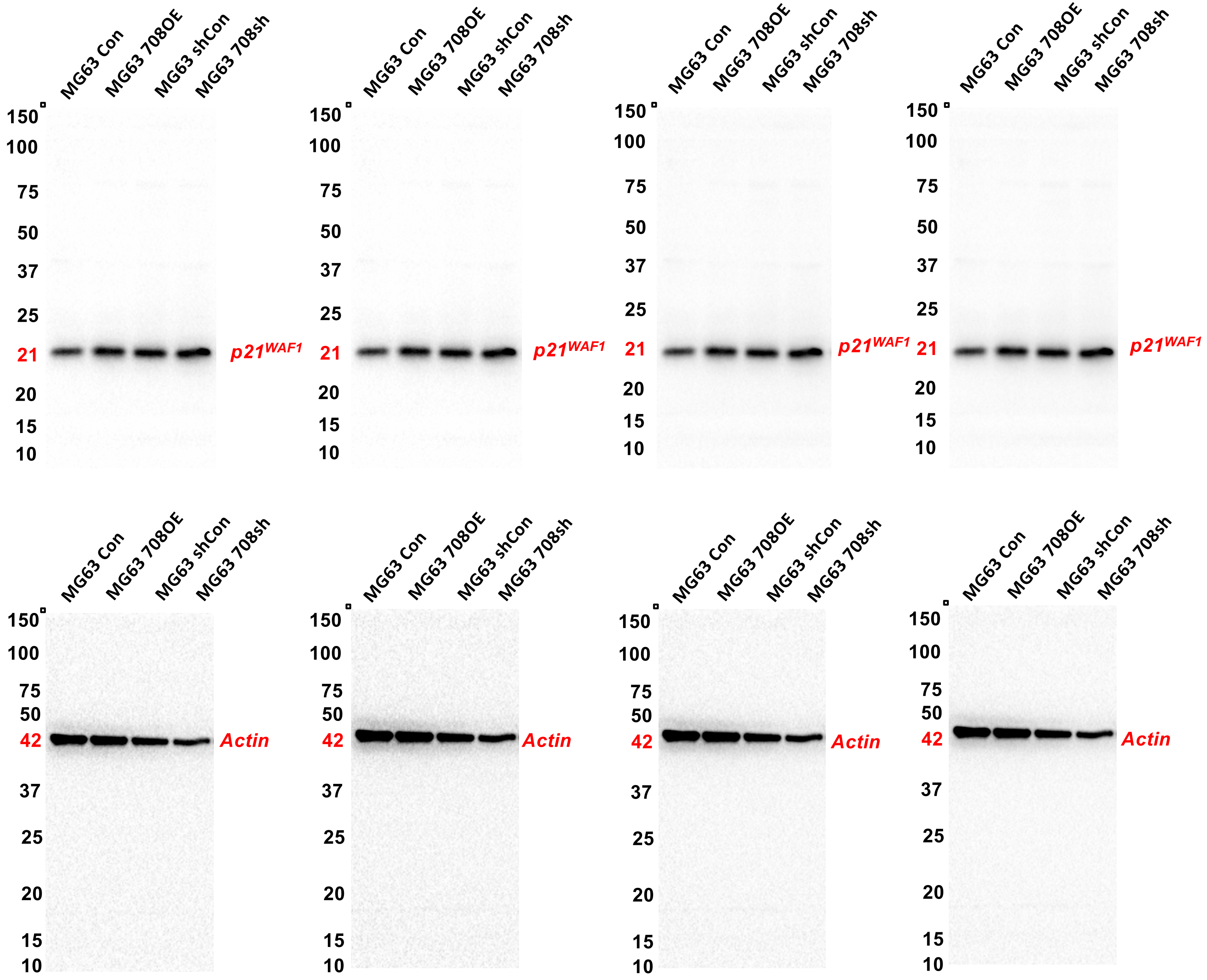

Western blots of protein of interest (*p21*^{WAF1}) and respective actin expression as given in Figure 7A with different exposure time

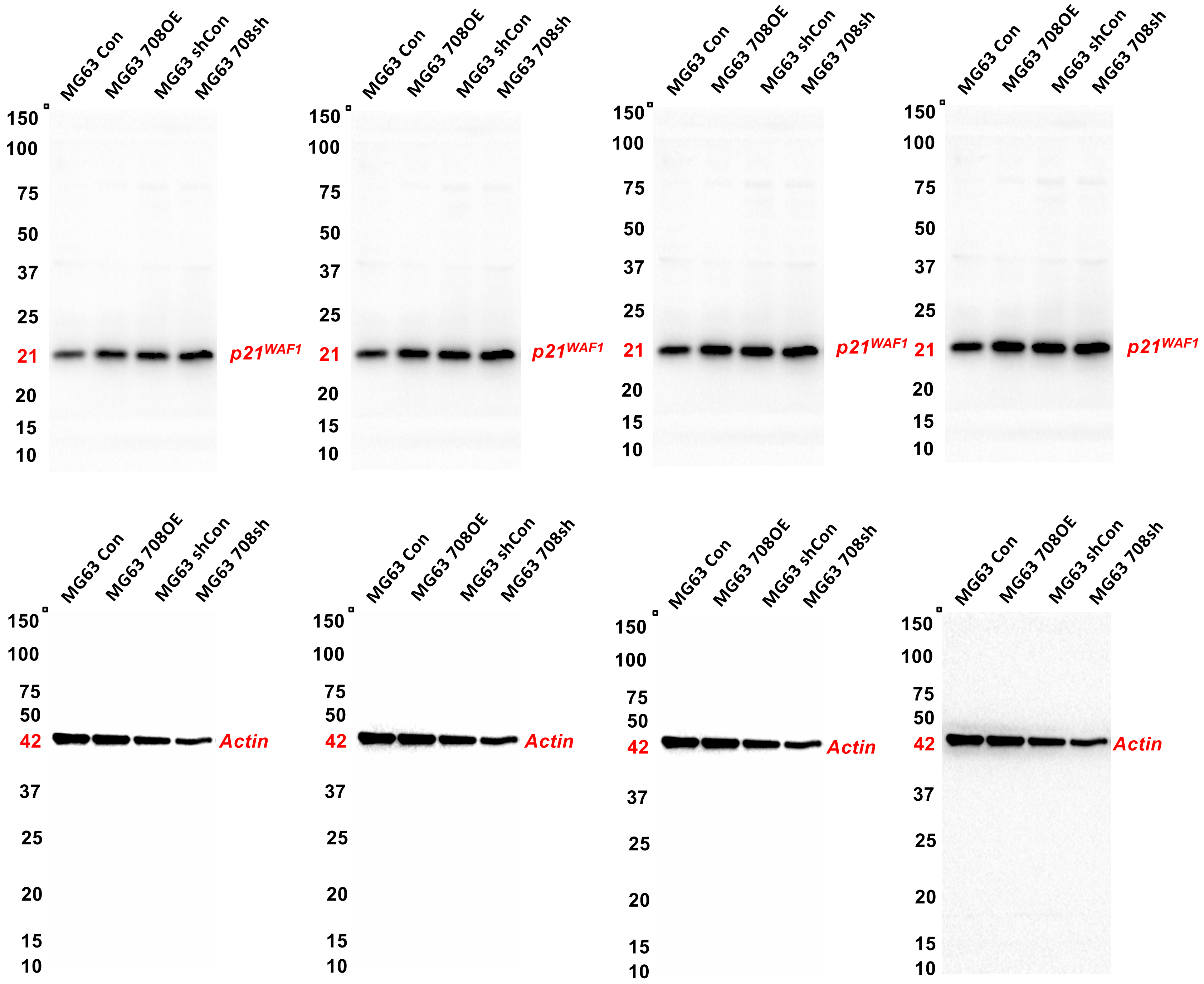

Western blots of protein of interest (*p21^{WAF1}*) and respective actin expression as given in Figure 7A with different exposure time

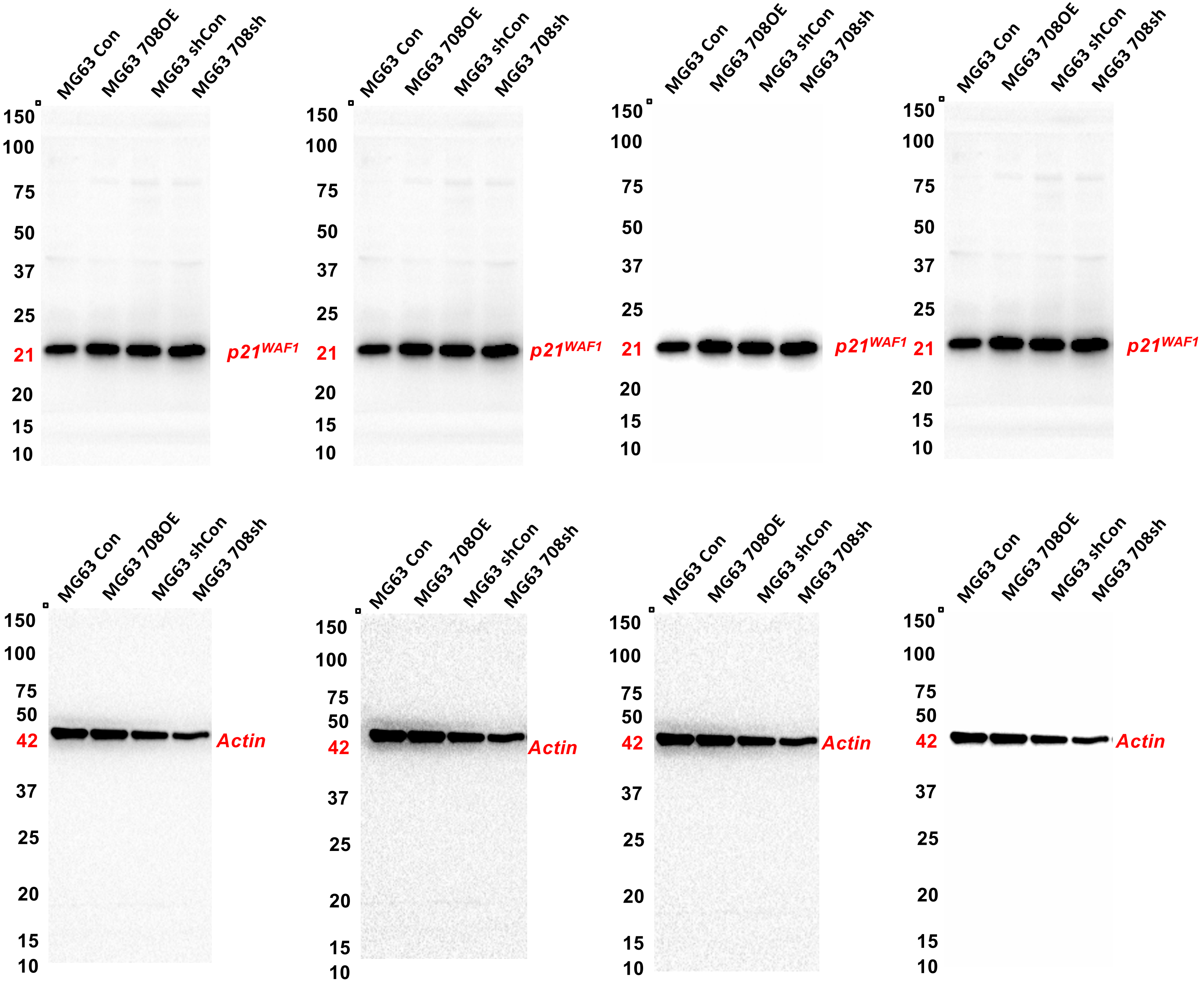

Western blots of protein of interest (*p21*^{WAF1}) and respective actin expression as given in Figure 7A with different exposure time

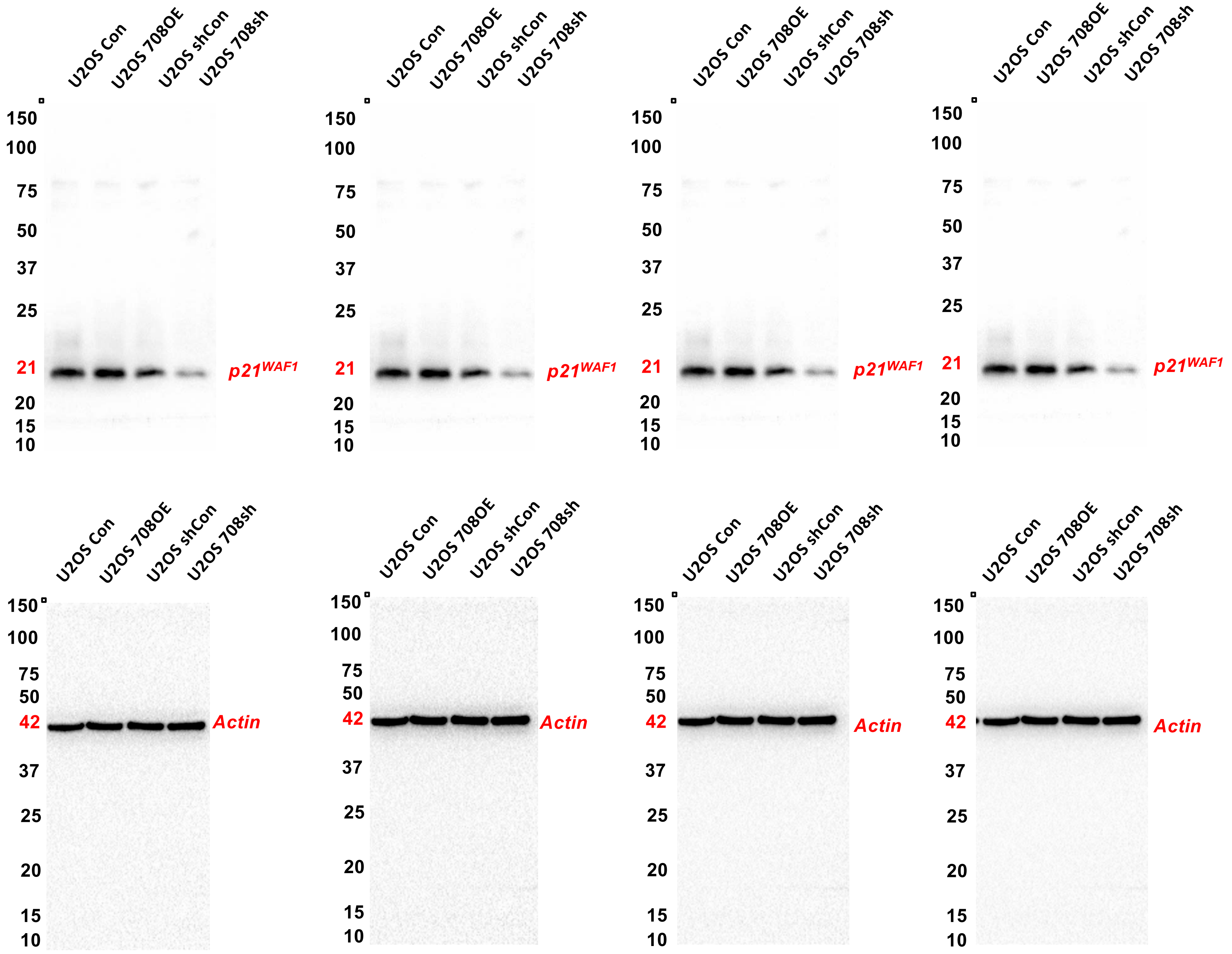
U2OS Con
U2OS 708OE
U2OS shCon
U2OS 708sh
150
100
75
50
37
25
21
20
15
10
p21WAF1
U2OS Con
U2OS 708OE
U2OS shCon
U2OS 708sh
150
100
75
50
37
25
21
20
15
10
p21WAF1
U2OS Con
U2OS 708OE
U2OS shCon
U2OS 708sh
150
100
75
50
37
25
21
20
15
10
p21WAF1
U2OS Con
U2OS 708OE
U2OS shCon
U2OS 708sh
150
100
75
50
37
25
21
20
15
10
p21WAF1
U2OS Con
U2OS 708OE
U2OS shCon
U2OS 708sh
150
100
75
50
42
37
25
20
15
10
Actin
U2OS Con
U2OS 708OE
U2OS shCon
U2OS 708sh
150
100
75
50
42
37
25
20
15
10
Actin
U2OS Con
U2OS 708OE
U2OS shCon
U2OS 708sh
150
100
75
50
42
37
25
20
15
10
Actin
U2OS Con
U2OS 708OE
U2OS shCon
U2OS 708sh
150
100
75
50
42
37
25
20
15
10
Actin

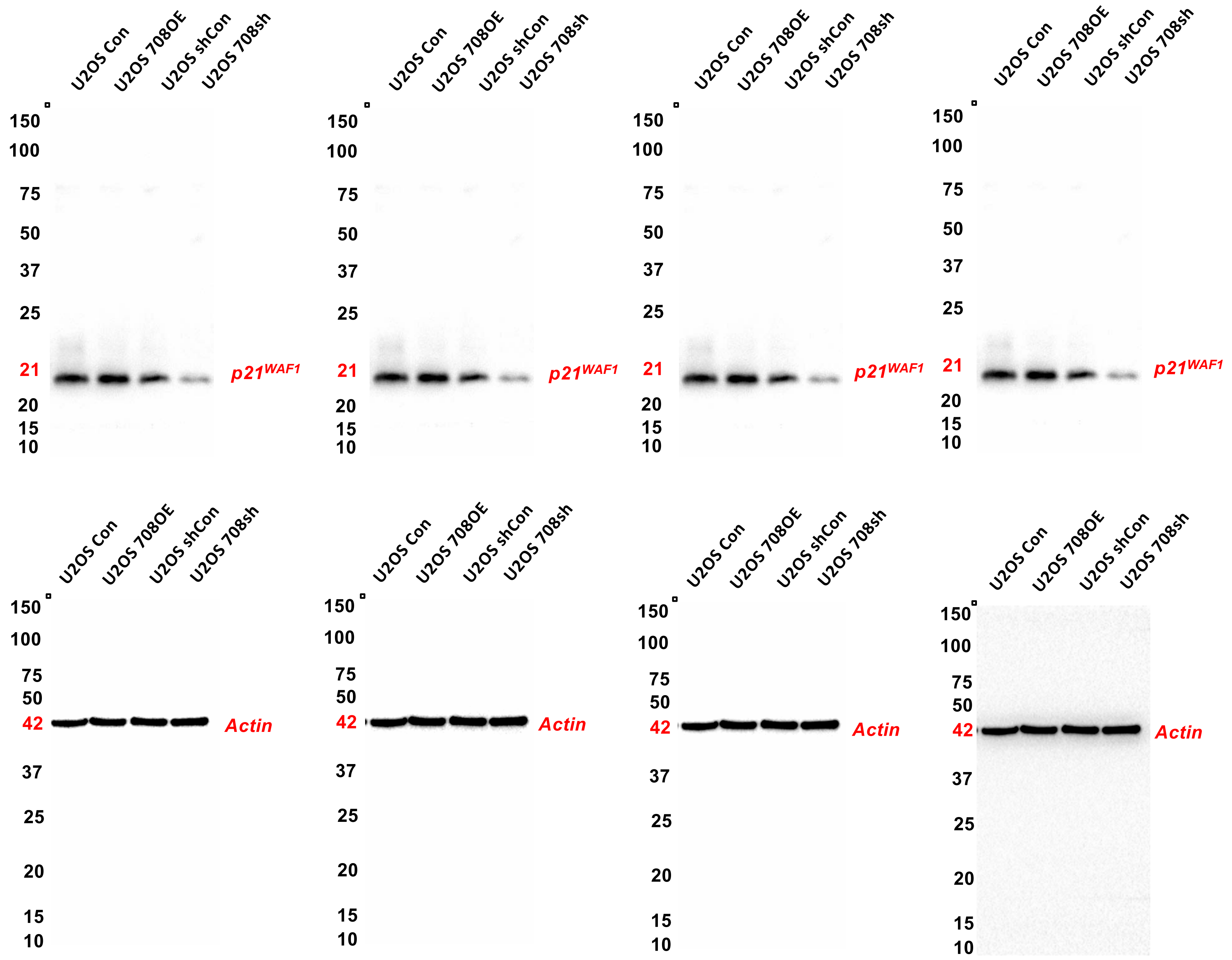

Western blots of protein of interest (*p21^{WAF1}*) and respective actin expression as given in Figure 7A with different exposure time

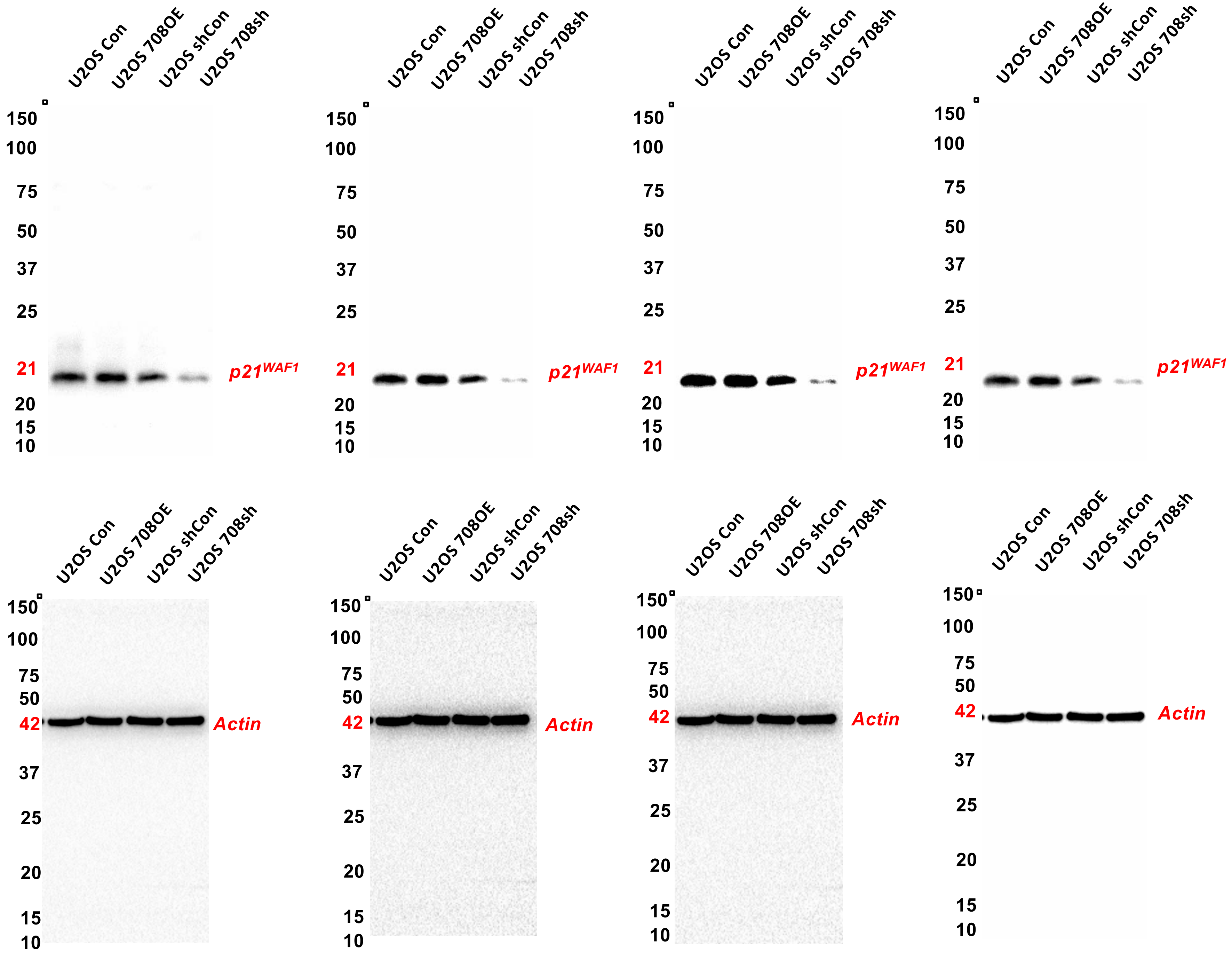

Western blots of protein of interest (*p21*^{WAF1}) and respective actin expression as given in Figure 7A with different exposure time
